# Anomalies in Network Bridges Involved in Bile Acid Metabolism Predict Outcomes of Colorectal Cancer Patients

**DOI:** 10.1371/journal.pone.0107925

**Published:** 2014-09-26

**Authors:** Sunjae Lee, KiYoung Lee, Seyeol Yoon, Jae W. Lee, Doheon Lee

**Affiliations:** 1 Department of Bio and Brain Engineering, KAIST, Yuseong-gu, Daejeon, Republic of Korea; 2 Department of Medical Informatics, School of Medicine, Ajou University, Yeongtong-gu, Suwon-si, Republic of Korea; 3 Neuroscience Section, Papé Family Pediatric Research Institute, Department of Pediatrics, Oregon Health and Science University, Portland, Oregon, United States of America; IRCCS Istituto Oncologico Giovanni Paolo II, Italy

## Abstract

Biomarkers prognostic for colorectal cancer (CRC) would be highly desirable in clinical practice. Proteins that regulate bile acid (BA) homeostasis, by linking metabolic sensors and metabolic enzymes, also called bridge proteins, may be reliable prognostic biomarkers for CRC. Based on a devised metric, “bridgeness,” we identified bridge proteins involved in the regulation of BA homeostasis and identified their prognostic potentials. The expression patterns of these bridge proteins could distinguish between normal and diseased tissues, suggesting that these proteins are associated with CRC pathogenesis. Using a supervised classification system, we found that these bridge proteins were reproducibly prognostic, with high prognostic ability compared to other known markers.

## Introduction

Colorectal cancer (CRC) is the third leading cause of cancer deaths worldwide, with 746,000 persons dying from this disease in 2012 [1]. Prognostic biomarkers would improve treatment strategies through risk stratifications [2]. To date, however, few indicators of patient prognosis have been identified, impeding the selection and timing of adjuvant therapy for at-risk patients.

Prognostic biomarkers should be mechanistically relevant to disease pathogenesis. Although current data-driven expression-signatures, where gene expression patterns are highly correlated with patient prognosis, have shown substantial prognostic ability, they have not revealed underlying mechanism and thus obscured proper therapeutic interventions [3]. Biological hypotheses have provided a priori evidence of mechanistic relevance [4], but existing targeted hypothesis-driven approaches are likely to miss out numerous genes related to the biological hypotheses, requiring new alternative approaches to find many hypothesis-relevant genes.

Bile acids (BAs) are carcinogenic [5], [6], with high-fat diets modulating BA homeostasis and altered levels of BAs leading to CRC pathogenesis. For example, a BA-supplemented diet in mice has been shown to induce CRCs directly, suggesting that BAs are carcinogenic [7]. However, although BAs lead to CRC pathogenesis, BAs were not utilized as practical markers. At in vivo levels, they were weak and indistinctive between patients with CRC and matched controls across studies [8] since changed BA levels by food intake are temporary and weak, thus difficult to detect. Anomalies in genes regulating cellular BA homeostasis are more of determinate factors to develop CRCs.

Proteins involved in the regulation of the homeostasis of not only BAs but all metabolites include metabolic sensors and metabolic enzymes. Metabolic sensors recognize the metabolic information during the regulation of homeostasis by detecting the levels of intracellular metabolites [9]–[11]. For example, the farnesoid X receptor (FXR, also known as NR1H4) detect the level of intracellular BAs, with this information utilized during the regulation of cellular BA homeostasis. Metabolic enzymes catalyze the reactions of metabolites, altering their intracellular levels. Anomalies in these sensors and enzymes would therefore alter BA homeostasis [12], [13] and ultimately affect CRC pathogenesis. For example, genetic defects in BA regulating enzymes or sensor proteins were found to lead to CRC pathogenesis [14], [15]. However, these genes also were not prognostic markers due to the low incidence of mutations in CRCs.

Interestingly, additional factors that are neither metabolic sensors nor enzymes were shown to modulate BA homeostasis [16]. As an alternative method of identifying reliable prognostic markers, we hypothesized that these factors may relay information on metabolic status between metabolic sensors and enzymes, functionally linking these two classes of molecules. These factors, called bridge proteins, may serve as reliable prognostic markers in patients with CRC, because anomalies in these proteins would disturb the delivery of metabolic information and the proper regulation of BA homeostasis. Current targeted approaches would be ineffective in probing relay proteins specifically between metabolic sensors and enzymes, due in large part to the lack of a method to quantify the relay degree of proteins. Systematic approaches, using information about known molecular interactions and the proteins connecting sensors and enzymes may identify and distinguish bridge proteins implicated in cellular signaling networks.

Here, we propose a network-based approach that identifies prognostic markers among proteins that play a critical role possibly linking sensors and enzymes of BA metabolism, relating to known biological hypothesis. These proteins, referred to as bridge proteins, can be assessed systematically based on information about molecular interactions recorded in several databases. To this end, we have defined a “bridgeness” metric, representing the degrees of connection between sensors and enzymes, and propose key bridge proteins as network markers for prognosis in patients with CRC. Using this “hypothesis-initiated” approach, we identified a set of markers that could better predict outcomes in patients with CRC than previously identified prognostic markers. A network-based investigation of biomarkers based on their bridgeness property may identify prognostic biomarkers implicated in cellular networks.

## Results

### Bridge networks and bridge proteins for bile acid metabolism

Our network-based approach identified 50 bridge proteins as reliable prognostic markers (**Table S1**). Top-ranked bridge proteins included peroxisome proliferator-activated receptor gamma, coactivator 1 alpha (PPARGC1A), hepatocyte nuclear factor 4 alpha (HNF4A), glycogen synthase kinase 3 beta (GSK3B), retinoid X receptor gamma (RXRG), caspase 8, apoptosis-related cysteine peptidase (CASP8), CREB binding protein (CBP), peroxisome proliferator-activated receptor alpha (PPARA), p53 (also known as TP53), E1A binding protein p300 (EP300) and retinoid X receptor alpha (RXRA). Notably, RXRA, forming a heterodimer with a BA sensor, FXR, participates in the regulation of BA homeostasis [17]. Also, p53 regulates BA homeostasis by linking between a BA sensor and BA enzymes, leading to abnormal BA accumulation by its defect [16], [18]. Likewise, some bridge proteins that function in regulating BA homeostasis are summarized in **Table S2**, showing evidence that bridge proteins, though they are computationally selected, may participate in the regulation of BA homeostasis.

**Figure 1:**
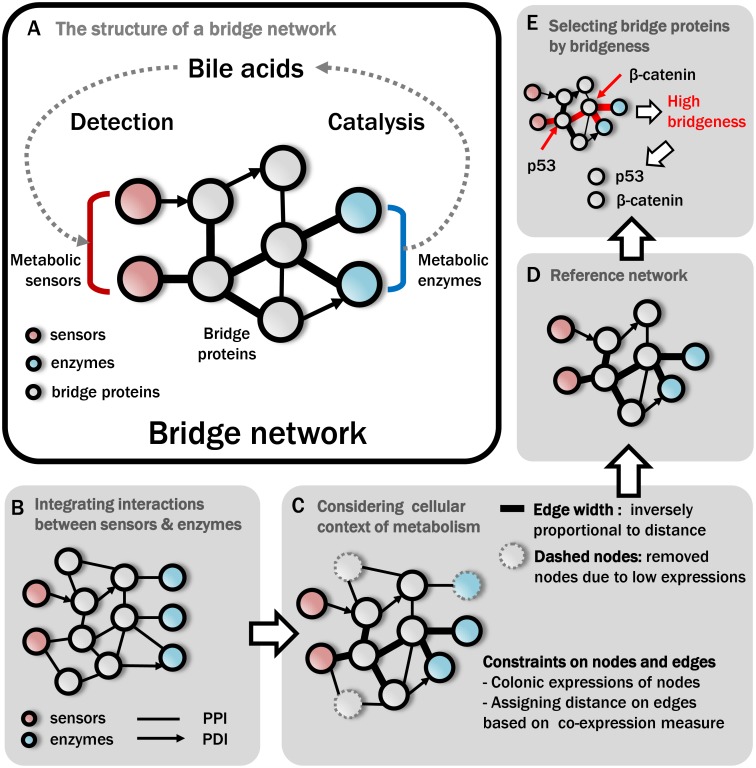
A bridge network for bile acid metabolism for determining bridge proteins. The overall process of the network construction is described in (**B–E**). (**A**) Structure of a bridge network, composed of a metabolic sensor (red), a metabolic enzyme (blue) and a bridge protein (gray). Metabolic enzymes catalyze the reactions of metabolites. Metabolic sensors detect the levels of intracellular metabolites. Bridge proteins link metabolic sensors and metabolic enzymes. (**B**) Integration of possible interactions between sensors and enzymes using protein-protein interactions (PPI) and protein-DNA interactions (PDI). Information on sensors and enzymes was collected from published studies and databases. (**C**) Imposing constraints on nodes and edges of an integrated network. (**D**) A final reference network to identify bridge proteins. (**E**) Selection of bridge proteins from the reference network by their bridgeness scores.

To investigate these bridge proteins, we constructed a reference network for BA metabolism (**Figure 1**), a network composed of metabolic sensors, metabolic enzymes and proteins linking sensors and enzymes. Pivotal bridge proteins that regulate given metabolic pathways were investigated by first integrating previous knowledge and interactome data. To date, 53 enzymes, including transporters, have been reported to be involved in BA metabolism and recorded in the EHMN database (**Table S3**) [19]. As detecting BAs and regulating their levels by altering downstream pathways for BAs, FXR has been found in vivo and in vitro to be a sensor for BAs [11]. Based on previous knowledge and the database, the sensor and enzymes were included in a BA bridge network. Large-scale interactome data from the databases, including HPRD [20] and TRANSFAC [21], were integrated to identify proteins that link sensors and enzymes (**Figure 1B**). We found that 10,805 genes or gene products were responsible for 110,741 interactions; of these gene products, we extracted only the sensors, enzymes and related intermediate proteins. All proteins responsible for direct and indirect interactions between sensors and enzymes were considered, with any intermediate protein being a possible bridge protein.

Constraints were subsequently imposed on both proteins and their interactions by considering the tissue-specific context of metabolism (**Figure 1C** **; Materials and Methods**). Despite abundant information on large-scale interactome data, there may be selection biases and tissue-specific variations. As a result of imposing constraints, we obtained a final reference network of 63,070 edges and 7,011 nodes, with sensors and enzymes constituting 23 nodes (**Figure 1D**, see **Figure S1** for the final reference network).

From the reference network, we selected bridge proteins, among intermediate proteins, that better link BA sensors and BA enzymes, using a “bridgeness” metric, assuming that the highly linking proteins critically regulate BA homeostasis through delivering metabolic information (**Figure 1E** **;** **Methods**). Compared with other existing centralities, including degree, closeness and betweenness centralities (see **Text S1**), our method was better able to focus on a particular protein’s connections in specific paths between sensors and enzymes, regardless of the connections in other unrelated paths on the network. As expected, locally dense proteins among paths between BA sensors and BA enzymes contribute significantly to the regulation of BA metabolism; thus, these proteins may be associated with CRC carcinogenesis. We therefore focused on the prognostic potential of bridge proteins with high bridgeness scores.

### Biological characteristics of bridge proteins

Before investigating their prognostic potentials, we examined the biological characteristics of bridge proteins that were computationally selected by bridgeness scores in CRCs. First, we identified expression patterns of bridge proteins embedded in CRCs; we examined discriminative patterns of bridge proteins at the transcriptomic level, using gene-expression profiles of CRC patients, as described previously [22]. Using univariate Student *t*-tests, we checked the ability of individual bridge proteins to distinguish between normal colon (*N* = 54) and primary CRC tissue samples (*N* = 186) at the transcriptomic level. Of the top-50 proteins, 42 (84%) were significantly discriminative (two sided *P*<0.01). Gene ontology enrichment analysis of these 42 proteins revealed that most were enriched in terms such as “regulation of transcription from RNA polymerase II promoter” and “transcription regulator activity”, which are related to regulatory roles in cellular processes (**Table S4**). They were also enriched in CRC pathogenic pathway-related terms, such as “canonical Wnt receptor signaling pathway” and “axin-APC-beta-catenin-GSK3B complex”, suggesting the relevance of these bridge proteins to CRC pathogenesis.

**Figure 2:**
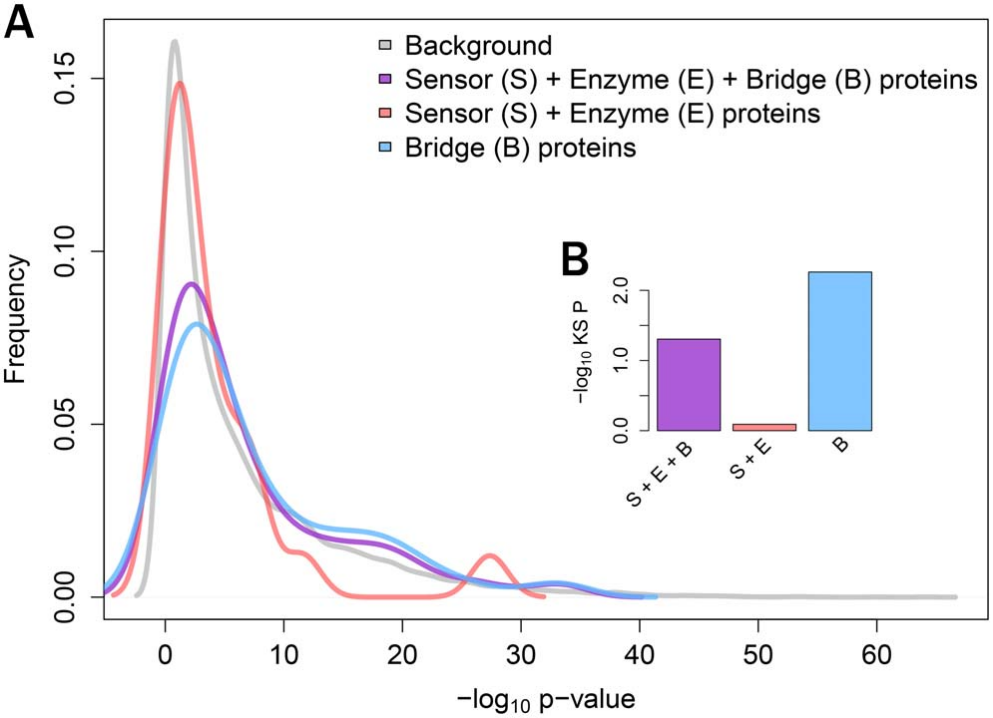
p-value distributions of components of a bridge network for bile acid metabolism. (**A**) p-value distributions of (i) sensor, enzyme and bridge proteins (S + E + B), (ii) sensor and enzyme proteins (S + E) and (iii) bridge proteins (B). (**B**) Comparisons of those p-value distributions with background p-value distribution. The statistical significance levels of shifted p-value distributions were determined by one-sided Kolmogorov Smirnov tests.

Next, we compared the p-value distributions of i) bridge proteins, ii) a sensor and an enzyme, and iii) a combined group of i) and ii) (**Figure 2**). Compared with the background distribution of p-values from overall gene products detected in a microarray (*N* = 12,752), the p-value distribution of the combined group was somewhat right-shifted (Kolmogorov-Smirnov (KS) test, one-sided *P* = 7.89×10^−2^). However, when we focused only on the bridge proteins, they showed high statistical significance in the KS test (*P* = 2.93×10^−3^), indicating that the discriminative power of bridge proteins, at the transcriptome level, was significantly greater than that of overall gene products in the microarray. Interestingly, sensor and enzyme proteins showed similar distributions relative to background (*P* = 0.812), indicating that sensor and enzyme proteins are less informative than bridge proteins in distinguishing between normal and diseased colon tissues.

We also investigated whether the top-50 bridge proteins are a feasible number of selections showing high statistical significance. We therefore compared the p-value distributions of selections with various numbers of bridge proteins, using the KS-test. The top-50 bridge proteins showed the lowest p-value on this comparison (**Figure S2**), with the statistical significance of selected bridge proteins being lower. Hence, we focused on the top-50 bridge proteins in further analysis. We also included other constraints used in network construction in a similar fashion (**Figure S3**).

**Figure 3:**
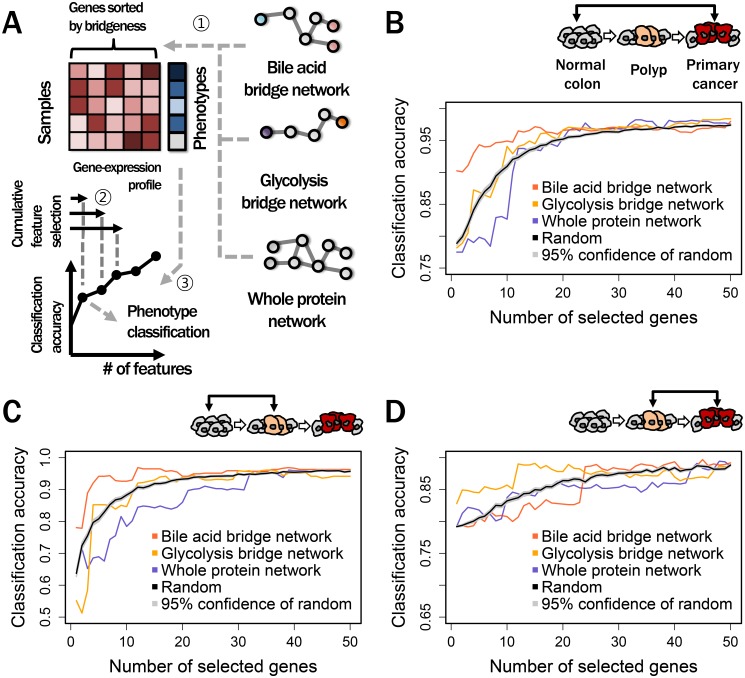
Multivariate analysis of bridge proteins from different networks. (**A**) Overall process of multivariate classifications using features from bridge proteins of different networks. After sorting bridge proteins by their bridgeness (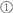), features were extracted cumulatively from top-ranked bridge proteins (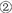). Samples were subsequently classified by cumulatively selected features and calculated classification accuracies (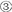). (**B**) Accuracies of classifications between normal colon and primary CRC tissues. For classifications, bridge proteins were obtained from (i) a bile acid bridge network (red), (ii) a glycolysis bridge network (yellow) and (iii) an whole protein network (purple). Classification accuracies were also calculated using randomly selected proteins (black) with 95% confidence intervals (gray) on the mean classification accuracies of repeated random selections (**C**) Accuracies of classifications between normal colon and polyp tissues. (**D**) Accuracies of classifications between polyp and primary CRC tissues.

We next compared the discriminative power of selected bridge proteins from different networks, through multivariate classification (**Figure 3A**) (**See detailed process in Materials and Methods**). The generated networks for comparisons were: (i) a bridge network developed from BA metabolism, (ii) a bridge network developed from glucose metabolism (i.e., glycolysis pathway) and (iii) a whole protein network without confining by sensors and enzymes in certain metabolic pathways. We also compared randomly selected proteins regardless of their interactions. Glycolysis was chosen for comparison to BA metabolism due to its relevance to common cancer progression [23], [24]. As expected, the discriminative power of a BA bridge network at the transcriptome level exceeded that of a glycolysis bridge network because glycolysis is not specifically involved in CRCs. The ability of components of the BA bridge network to classify a sample as normal colon or primary CRC tissue (**Figure 3B** **)** largely exceeded that of randomly selected gene products. In contrast, components of other networks, including that involved in glycolysis, were equal to or barely exceeded randomly selected gene products in discriminative ability. That is, only gene expression levels of bridge proteins selected from a BA bridge network according to bridgeness were informative in distinguishing between normal colon and CRC.

We then examined CRC stage-specific expression patterns of selected bridge proteins. Most sporadic CRCs develop from normal colon via adenomatous polyps, with the sequence involving accumulated genetic anomalies in a stepwise manner [25]. To identify stage-specific variations in bridge proteins, we performed multivariate classifications between normal colons and adenomatous polyps and between polyps and primary CRCs. We found substantial variations in gene expressions of bridge proteins between normal colons and polyps (**Figure 3C and D**). Namely, bridge proteins associated with BA metabolism varied substantially during early stages of CRC pathogenesis, suggesting that these bridge proteins may be initiators of CRC tumorigenesis. We also found that bridge proteins from BA metabolism and glycolysis exhibited inverse patterns between polyps and primary CRCs, showing weaker, but substantial, variations during later stage of CRC pathogenesis, as if these changes were followers of CRC development (**Figure 3C and D**). Together, these findings showed that bridge proteins from BA metabolism and glycolysis behaved commutatively during CRC progression.

Furthermore, using pathway enrichment tests, we observed other meaningful biological characteristics of bridge proteins. Bridge proteins involved in BA metabolism were enriched in CRC-related pathways, including the Wnt (KEGG ID: hsa04310; false discovery rate-adjusted, hypergeometric *P* = 4.47×10^−5^), CRC (KEGG ID: hsa05210; *P* = 2.80×10^−5^) and common cancer (KEGG ID: hsa05200; *P* = 6.94×10^−10^) pathways (**Table S5**). This finding indicates that most bridge proteins are involved in CRC pathogenesis-related pathways and have the potential to promote CRCs through these pathways. Thus, characteristics determined from discriminative patterns and enrichment tests indicate that bridge proteins selected by bridgeness are associated with CRC pathogenesis.

### Potential of bridge proteins as prognostic markers

**Figure 4:**
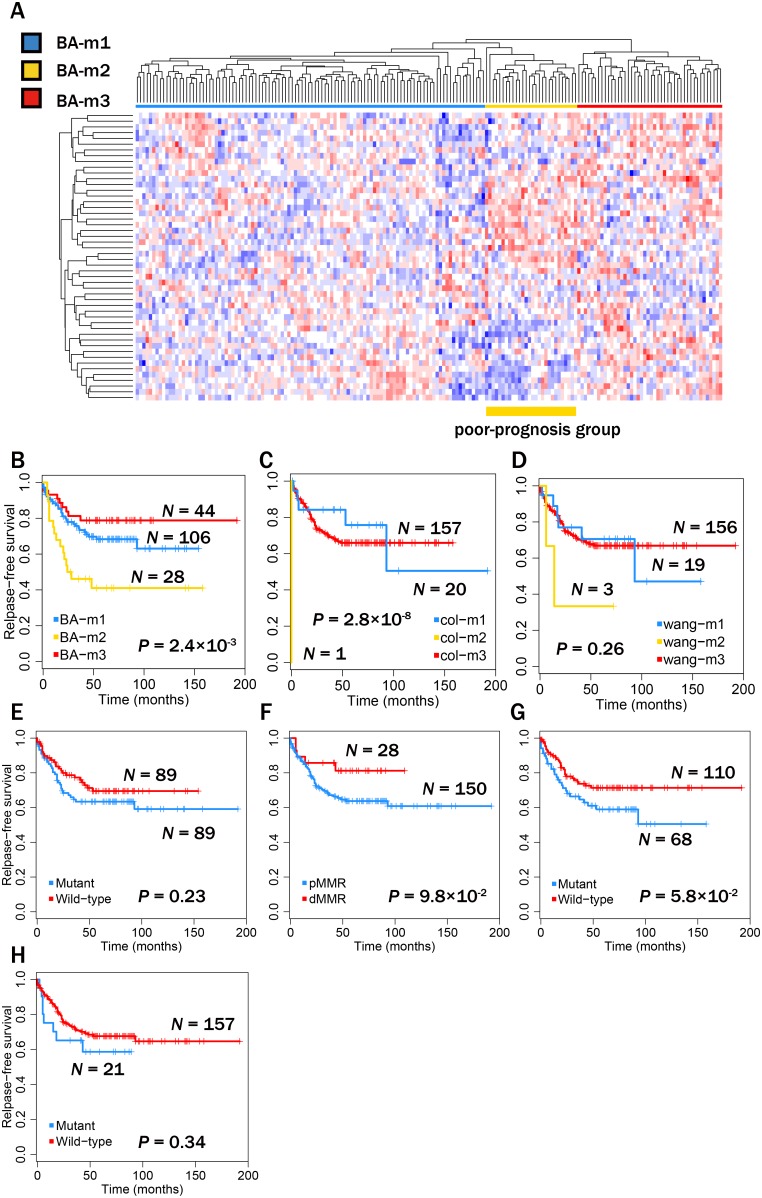
Identification of the prognostic ability of markers. Their prognostic ability was examined using a dataset of tissue samples from patients with CRC [26]. (**A**) Heatmap of CRC tumor samples with subgroups classified by the expression patterns of bridge proteins: BA-m1 (blue), BA-m2 (yellow) and BA-m3 (red). Prognostic ability was assessed by Kaplan-Meier survival analyses. The BA-m2 group showed the poorest prognosis. (**B**) Prognostic ability of our bridge proteins. (**C**) Prognostic ability of the ColoPrint gene set [28], with subgroups classified as col-m1 (blue), col-m2 (yellow) and col-m3 (red). (**D**) Prognostic ability of the Wang et al. signature gene set [27], with subgroups classified as wang-m1 (blue), wang-m2 (yellow) and wang-m3 (red). (**E**) Prognostic ability of p53 mutation status, mutant and wild-type. (**F**) Prognostic ability of mismatch repair gene (MMR) status, deficient (dMMR) and proficient (pMMR). (**G**) Prognostic ability of KRAS mutation status, mutant and wild-type. (**H**) Prognostic ability of BRAF mutation status, mutant and wild-type.

To assess the prognostic ability of computationally-selected bridge proteins, we assessed their expression patterns in patients classified as having a good or poor prognosis. First, we clustered patients in an unsupervised way, based on similarities of expression patterns, and compared survival outcomes among patients in clusters. Total 178 patients from previous dataset [26] were clustered into three subgroups using a hierarchical clustering algorithm: BA-m1 (*N* = 106), BA-m2 (*N* = 28) and BA-m3 (*N* = 44) (**Figure 4A**). The Kaplan-Meier method with the log-rank test showed that among three subgroups of patients, the relapse-free survival was significantly different, indicating their substantial prognostic potential (*P* = 2.37×10^−3^) (**Figure 4B**). Then, we assessed the prognostic potential of other known expression-signature markers in the same way. Using expression patterns of genes selected in Wang et al [27] and ColoPrint [28], we classified patients into three subgroups and compared survival outcomes among their subgroups (ColoPrint’s subgroups: col-m1 (*N* = 20), col-m2 (*N* = 1) and col-m3 (*N* = 157); Wang’s subgroups: wang-m1 (*N* = 19), wang-m2 (*N* = 3) and wang-m3 (*N* = 156)). As a result, subgroups of patients clustered by ColoPrint’s genes can distinguish between good and poor prognoses (*P* = 2.75×10^−8^), though just a single patient found in the poorest prognosis group (col-m2), but Wang’s genes were not prognostic (*P* = 0.258) (**Figure 4C and D**). In addition, known molecular markers, including p53 mutations (*P* = 0.233), mismatch repair gene status (*P* = 9.8×10^−2^), KRAS mutations (*P* = 5.75×10^−2^), and BRAF mutations (*P* = 0.338), were not also substantially prognostic in this dataset (**Figure 4E–H**).

**Figure 5:**
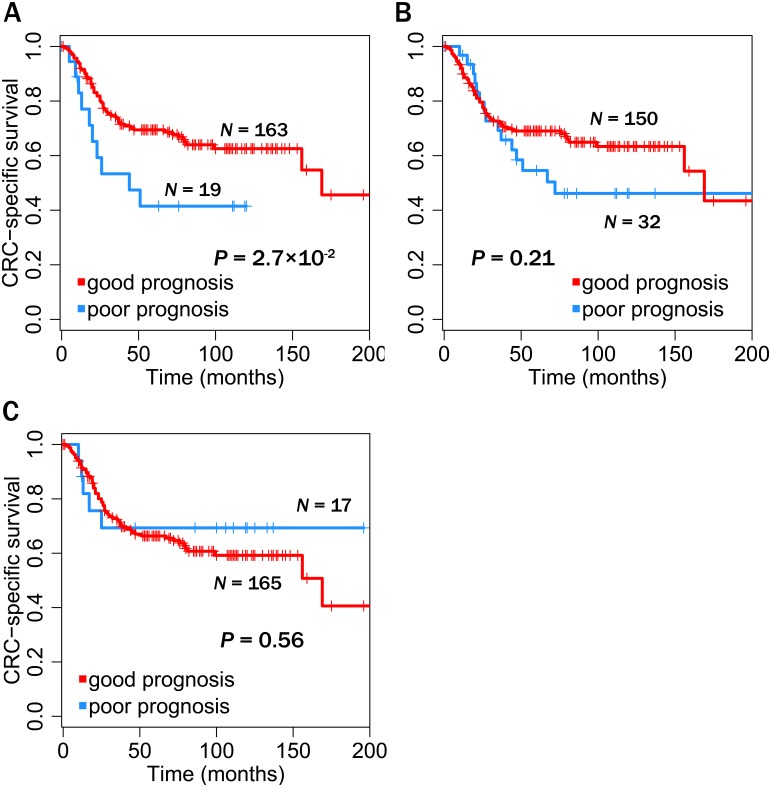
Identification of the prognostic reproducibility of markers. Their prognostic ability was examined in an independent test data [22] by supervised classifications and thus confirmed their prognostic reproducibility. (**A**) Prognostic ability of our bridge proteins (**B**) Prognostic ability determined by the ColoPrint gene set in reference [28] (**C**) Prognostic ability determined by the Wang et al. gene set in reference [27].

To assess the prognostic reproducibility of these bridge proteins and other expression-signature markers, we then classified patients in an independent dataset [22] as having good or bad prognoses, through a supervised classification system, using previous dataset [26] as the training dataset (**Figure 5**). Patients in the test data were classified, using their expression levels, based on correlation coefficients to mean expression levels of poor-prognosis-group patients in the training data, like previously performed [29]; we assigned patients into a poor-prognosis group if their correlation coefficients were high. We obtained thresholds of correlation coefficients to decide poor-prognosis patients with the highest statistical significance, through cross-validation procedures on the training data (See Materials and Methods). Noteworthy, patients in the test data can be significantly distinguished between good and poor prognoses when we used expression levels of bridge proteins as features for correlation coefficients; survival outcomes, i.e., CRC-specific survivals, of classified groups by the bridge proteins were significantly different when the Kaplan-Meier method with the log-rank test was used (P = 2.70×10^–2^) (**Figure 5A**). Other expression signatures, including ColoPrint (P = 0.210) and Wang’s (P = 0.558) (**Figure 5B and C**), were not prognostic in the independent test dataset, suggesting that only bridge proteins were reproducibly prognostic. These results underline the potential and reliability of bridge proteins as prognostic markers.

## Discussion

By investigating genes involved in the regulation of BA homeostasis, this study has identified numerous genes for prognostic biomarkers of CRC, with showing mechanistic relevance to CRC pathogenesis. Although various prognostic biomarkers have been proposed based on biological hypotheses [4], these biomarkers have shown limited clinical usefulness. The hypothesis, that BAs play pivotal roles in CRC, provides clues to understanding the pathogenesis of this disease. However, rather than focusing on BAs themselves, we focused on the genes involved in regulating BA metabolism by linking metabolic sensors and metabolic enzymes. Based on a devised metric, “bridgeness”, numerous bridge proteins were selected from a reference, or bridge, network, and their prognostic abilities were analyzed. Bridge proteins could distinguish between normal and diseased tissues and are therefore relevant to the pathogenesis of CRC. These bridge proteins had greater and reproducible prognostic ability, as shown by statistical significance, than previously identified prognostic markers, suggesting that they are reliable prognostic markers in patients with CRC.

Interestingly, however, neither sensor nor enzyme proteins could significantly distinguish between normal colon tissue and CRC, a finding that may result from the housekeeping roles of these sensor and enzyme proteins for cell survival. Cells lack proteins with molecular functions similar to those of most of these sensor and enzyme proteins; thus, defects in their expression would have detrimental effects on cellular functions. Thus, evolutionarily, genetic anomalies in bridge proteins may have survival advantages over anomalies in sensor and enzyme proteins. Indeed, some bridge proteins, including caspase 8, apoptosis-related cysteine peptidase (CASP8), p53 and catenin (cadherin-associated protein) beta 1, 88 kDa (CTNNB1, also known as β-catenin), showed high mutational frequencies in CRC samples, whereas sensor and enzymes proteins for BA metabolism did not [30]. This evolutionary pressure, including during CRC tumorigenesis, would accelerate the acquisition of anomalies by bridge proteins.

In previous studies, notably, one bridge protein, STK11, was shown to have particular mechanistic potential to promote colorectal tumorigenesis [31]–[33]. STK11 has been associated with Peutz-Jeghers syndrome (PJS), a condition that enhances the formation of gastric adenomatous polyps and hepatocellular carcinoma [31]. In most PJS patients, one allele of STK11 is mutated, causing multiple gastric adenomatous polyps or hepatocellular carcinoma [32], [33]. Similarly, STK11 may have the mechanistic potential to promote colorectal tumorigenesis. Other bridge proteins may also have prognostic value in CRC pathogenesis.

STK11 is also associated with energy metabolism, either alone or by interacting with AMPK, making it a potential bridge protein involved in the regulation of energy metabolism [34], [35]. Among the other bridge proteins involved in energy metabolism are PPARGC1A, GSK3B, PPARA, peroxisome proliferator-activated receptor gamma (PPARG), solute carrier family 2 (facilitated glucose transporter) member 4 (SLC2A4, also known as GLUT4), glyceraldehyde-3-phosphate dehydrogenase (GAPDH), and lactate dehydrogenase A (LDHA), all important regulators of or enzymes involved in energy metabolism. Thus, their molecular functions may explain the activities of BAs that increase energy expenditure [36]. Assessments of the molecular functions of bridge proteins may provide novel insights on their as yet unidentified roles in BA homeostasis.

Despite bridge proteins showing prognostic potential, BA bridge networks show limited ability to identify other known CRC-susceptibility genes. For example, we found that a BA bridge network was unable to identify several well-known CRC-susceptibility genes, such as APC, KRAS, and BRAF. Inaccuracies originating from large-scale interactome data could impede in-depth analysis of bridge networks. Also, the interrelations of metabolic pathways, such as lipid, cholesterol, and glucose metabolism, would extend the ability to investigate all risk factors for CRC pathogenesis. This approach could also be applied to other diseases vulnerable to metabolic anomalies, including obesity, type-2 diabetes and Alzheimer’s disease once metabolic sensors, enzymes and proper interactome data are generated for these diseases. The determination of proper and accurate bridge networks for metabolic pathways can allow the identification of disease-susceptibility genes and their clinical use as prognostic markers.

In summary, we found that bridge proteins, which are involved in the regulation of BA metabolism, have prognostic potential in patients with CRC. Despite their potential to promote CRC pathogenesis, bridge proteins had not been systematically investigated in previous studies. Based on a devised metric for “bridgeness”, we computationally selected bridge proteins from a reference network and examined their prognostic potential in CRC. We also tested whether differences in their discriminative expression patterns in normal colon and CRC made them relevant to CRC pathogenesis. The findings indicate that bridge proteins involved in the regulation of BA metabolism may be reliable prognostic markers for CRC patients.

## Materials and Methods

### Bridge network construction

The reference network for BA metabolism consisted of metabolic sensors, metabolic enzymes and proteins interacting with both. The selected BA sensor was FXR and the BA enzymes were those designated in the EHMN human metabolic network database as enzymes involved in the “bile acid biosynthesis” pathway [19]. Possible interactions between the sensor and the enzymes were integrated using protein-protein interactions (PPI) described in the HPRD human protein information database [20] and protein-DNA interactions (PDI) from the commercial TF binding site database, TRANSFAC (Ver. 11.1) [21]. PPIs were regarded as bidirectional interactions and PDIs as unidirectional interactions from TFs to target genes. Next, we imposed constraints on the integrated network. On edges, we assigned distance values using a co-expression measure (i.e., the distance *d_ij_* between genes *i* and *j* was defined as *d_ij_* = 1–*r^2^_ij_* where *r_ij_* is Pearson’s correlation coefficient for the correlation in expression between genes *i* and *j*). Co-expression, defined as the functional relationship of a pair of proteins [37], was calculated using recently published FACS-sorted cell expression profiles from 52 patients with CRC [38], obtained from the public gene expression profile database, GEO (ID: GSE39397). On nodes, we imposed constraints regarding colonic gene expression. Using human whole-tissue gene expression data obtained from the public database, BioGPS [39], we determined the colonic expression of individual genes and compared the colonic and tissue-wide expression of each (total 176 samples with 84 tissue types; two samples for a colon tissue). If the average ratio of colonic to tissue-wide expression was lower than our criterion, that gene was removed. The criterion for gene removal was determined by comparing the p-value distribution of 50 bridge proteins with a background p-value distribution, as described in Results (**Figure S3**). In those comparisons, a 40th percentile cutoff produced the highest significance of the shifted p-value distribution.

Similarly, we constructed bridge networks relative to glycolysis and all proteins without specification for network comparisons. All the processes were identical to those used to construct the BA bridge network, except for the selection of metabolic sensors and enzymes. For glycolysis, we selected the metabolic sensors egl nine homolog 2 (C. elegans) (EGLN2, also known as PHD1), egl nine homolog 1 (C. elegans) (EGLN1, also known as PHD2), egl nine homolog 3 (C. elegans) (EGLN3, also known as PHD3) and hypoxia inducible factor 1 alpha subunit inhibitor (HIF1AN, also known as FIH). Their sensing of glycolysis metabolites was determined in vitro and in vivo [40]. Metabolic enzymes for glycolysis pathway were obtained from the “glycolysis and gluconeogenesis” pathway in the EHMN database [19]. For the whole protein network, we regarded metabolic sensors and enzymes as all the genes in the network in order to avoid specification by certain types of metabolism.

### Bridgeness score

The bridgeness metric of a gene *i* with a set of sensors *S* and a set of enzymes *T* was calculated as:
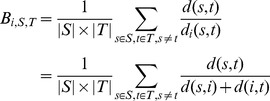
where *d(s,t)* represents the distance of the shortest path between a sensor *s* and an enzyme *t*, and *d_i_(s,t)* represents the distance of the shortest path between node *s* and node *t* via node *i*. If gene *i* in the network is far from the shortest path between sensors and enzymes (i.e., *d_i_(s,t)*≫*d(s,t)*), then the addend tends to zero. Therefore, the bridgeness of gene *i* would be high if it is located near the shortest paths between sensors and enzymes, thus avoiding unrelated paths in cellular signaling networks. All calculations of network features and bridgeness were determined using R language and the *igraph* package [41].

### Univariate and multivariate analysis

For univariate and multivariate analyses, we used a gene expression profile from CRC patients [22], which we obtained from the GEO database (ID: GSE41258). This dataset includes gene expression in 54 normal colons, 49 adenomatous polyps and 186 primary CRC tissue samples. Before using gene expression profiles to distinguish among tissue types, we performed gene-wise normalization on the profile using *Z* score transformation. In univariate analysis, the ability of each gene’s expression to distinguish normal colon and primary CRC tissues was assessed by Student’s *t*-test. We also calculated the statistical significance of the shifted p-value distribution of genes of interest against a background p-value distribution using the two-sample Kolmogorov-Smirnov one-sided test with the support of R package, *stats*. In multivariate analysis, we identified a bridge protein’s discriminative ability, at the transcriptome level, using a logistic regression model with the support of java machine learning API, *Weka* [42]. Multivariate features were cumulatively selected from top-ranked bridge proteins of networks. The ability of each selection to classify samples as normal colon or primary CRC was evaluated using the five-fold cross-validation method with five repeats. The ability of each to distinguish between normal colon and polyp tissues, and between polyps and primary CRCs tissues, was assessed using the same features. We also simultaneously evaluated randomly selected proteins with an equal number of features. At each evaluation step, classification accuracy (i.e. accuracy =  
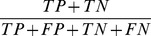
) was measured and averaged after five repeats. In assessing features of randomly selected proteins, we calculated the mean classification accuracy after 100 repeats of selections and afterward calculated a 95% confidence interval of mean classification accuracies.

### Survival analysis

First, the prognostic ability of bridge proteins was determined using related information from the Marisa et al. dataset [26] in the GEO database (ID: GSE39582). Information was available about gene expression; CRC recurrence-free survival event and time; treatment status; molecular marker status, including p53, KRAS, and BRAF mutations; and mismatch repair gene status. In this dataset, we used 178 tumor samples of patients to assess the prognostic ability; samples from treated patients or with missing information about survival outcomes or molecular status were excluded, avoiding unexpected effects of treatment on survival outcomes or unknown information. Identifying prognostic ability, we clustered patients, based on Euclidean distances between gene expressions of patients, by an unsupervised hierarchical clustering algorithm and measured the difference of survival outcomes among the patient clusters by the Kaplan-Meier method with the log-rank test. To compare prognostic abilities with other gene-expression signature markers, we used two gene sets, Wang’s (*N* = 21) [27] and ColoPrint (*N* = 15) [28], and assessed their prognostic ability using their expression profiles from patients with CRC. In the two comparative gene sets [27], [28], we only utilized genes detected in microarray data that we applied.

We also assessed the prognostic reproducibility of bridge proteins through a supervised classification system (**Figure S4**). In this classification system, the previous dataset [26] were used as a training data and the Sheffer et al. dataset [22] were used as an independent test data during supervised classifications. Total 182 tumor samples of patients from the Sheffer et al. data were used, after excluding samples that were not used in the original study [22]. This dataset contains information about gene expression and CRC-specific survival event and time. Performing supervised classification, we first determined a patient group with the poorest prognosis from the training data, after clustering patients by a hierarchical clustering and comparing survival probabilities among patient clusters. Referencing mean expression levels of patients in the poorest prognosis group (i.e. BA-m2 in **Figure 4A**) as a criterion, we classified patients of the test data into poor prognoses if their correlations of gene expressions with the reference expression levels are higher than a threshold, like existing study [29]. We calculated the correlations based on Pearson’s correlation coefficients. A threshold of a correlation coefficient deciding prognosis was obtained through cross-validated procedures using the training data [26]. In this data set, we performed supervised classifications through five-fold cross-validations with various thresholds and selected the best threshold that can distinguish patients into a good or poor prognosis group with the most statistical significance. The statistical significance was measured by the Kaplan-Meier method with the log-rank test. We repeated cross-validations 100 times and averaged best thresholds in all repeats as a final threshold to use. Based on the final threshold, at last, we classified patients in an independent test data with learning a training data. We performed supervised classifications by other expression signatures in a similar way. All the statistical analyses, including Kaplan-Meier survival analysis, were performed by R packages.

## Supporting Information

Figure S1 **A final reference network for bile acid metabolism.** This network is composed of a metabolic sensor (red), metabolic enzymes (blue) and interplay proteins (the outer layer of the largest circle). The Top-50 bridge proteins (black) are also shown. The edges representing the shortest paths between a sensor or an enzyme and a top-50 bridge protein are underlined (red edges).
(TIF)

Figure S2 **p-value distributions of bridge proteins with varying numbers of selections.** They stand for p-value distributions of the (**A**) top-10, (**B**) top-20, (**C**) top-30, (**D**) top-40, (**E**) top-50, (**F**) top-60, (**G**) top-70, (**H**) top-80, (**I**) top-90, and (**J**) top-100 bridge proteins. Statistical significance was highest for the top-50 bridge proteins when the shifted degrees of background (gray) and selected (blue) p-value distributions were measured using the one-sided Kolmogorov-Smirnov test.
(TIF)

Figure S3 **p-value distribution of bridge proteins according to imposed constraints.** (**A**) without node or edge constraints, (**B**) without node constraints, (**C–F**) with node constraints of (**C**) 10%, (**D**) 20%, (**E**) 30%, and (**F**) 40% removal criteria. Node removals within 40% were the most feasible for network construction.
(TIF)

Figure S4 **An overview of a supervised classification system.** The pipeline of supervised classification system was demonstrated. We used Marisa et al. dataset as a training data and Sheffer et al. dataset as a test data, after filtering out samples of patients in undesired conditions (1). Supervised classifications were based on correlations of gene expressions between the reference from the training data and samples from the test data. To select the threshold of correlation coefficients for deciding prognosis, we performed cross-validation procedure; we repeated five-fold cross-validation 100 times and averaged best threshold in all repeats (2). Based on the threshold obtained, we classified patients in the test data (3) and compared survival outcomes among classified patient groups, having a good or poor prognosis, through the Kaplan-Meier method with the log-rank test.
(TIF)

Table S1 **Top-50 bridge protein information.** We showed statistics of each bridge protein about discriminative power (T-score and T-test P) using datasets of Sheffer et al.
(DOCX)

Table S2 **Evidence of bridge proteins involved in the regulation of bile acid homeostasis.** Shown was previous literature that identified bridge proteins as being involved in the regulation of bile acid homeostasis. In the second column, we provided literature with definitive evidence that defects of some bridge proteins cause abnormal changes of bile acid levels. In the third and fourth columns, we provided literature with indirect evidence: studies in the third column showing that bridge proteins were regulated by or co-activated with a bile acid sensor; studies in the fourth column showing that bridge proteins regulated enzymes in bile acid metabolism.
(DOCX)

Table S3 **Sensor and enzyme proteins in bile acid or glucose metabolism.**
(DOCX)

Table S4 **Enriched GO terms under corrected p-value<0.01.**
(DOCX)

Table S5 **Enriched KEGG non-metabolic pathways under FDR-adjusted hypergeometric p-value<0.01.**
(DOCX)

Dataset S1 **A source code and a dataset for extracting bridge proteins involved in bile acid metabolism.** Performing a source code with a dataset will provide an output file showing top-50 bridge proteins we used.
(ZIP)

Dataset S2 **A source code and a dataset for survival analyses in** **Figure 4** **and** **5** **.** Performing a source code with a dataset will provide figures shown in our manuscripts.
(ZIP)

Text S1 **Characteristics of bridgeness scores.**
(DOCX)
