# Supplementary figures and images for: Anomalies in Network Bridges Involved in Bile Acid Metabolism Predict Outcomes of Colorectal Cancer Patients
Source: PLoS One. 2014 Sep 26;9(9):e107925. doi: 10.1371/journal.pone.0107925 (PMC4178056; doi:10.1371/journal.pone.0107925)

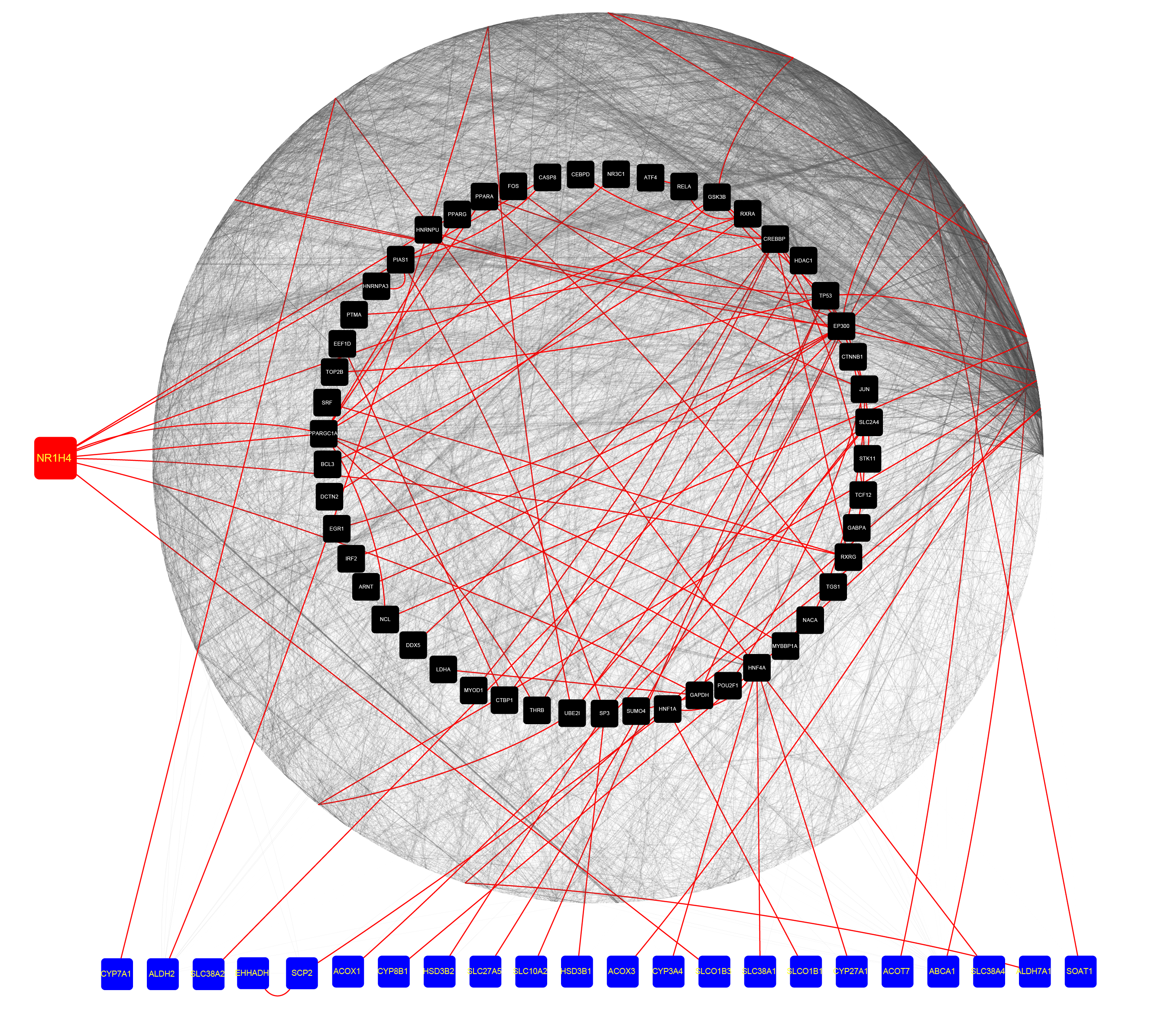

Supplement: Figure S1 — A final reference network for bile acid metabolism. This network is composed of a metabolic sensor (red), metabolic enzymes (blue) and interplay proteins (the outer layer of the largest circle). The Top-50 bridge proteins (black) are also shown. The edges representing the shortest paths between a sensor or an enzyme and a top-50 bridge protein are underlined (red edges). (TIF) [file pone.0107925.s001.tif]

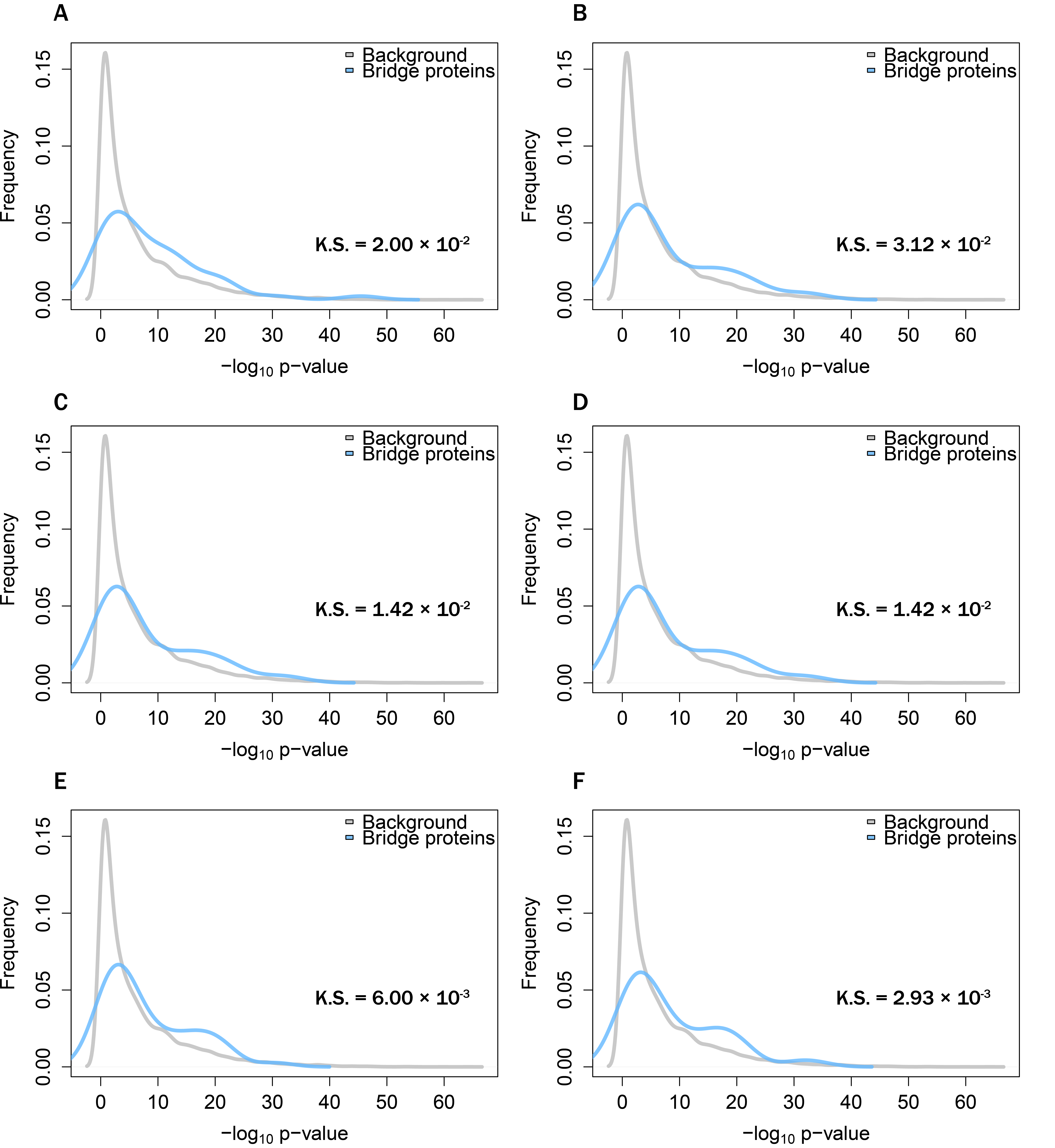

Supplement: Figure S3 — p-value distribution of bridge proteins according to imposed constraints. (A) without node or edge constraints, (B) without node constraints, (C–F) with node constraints of (C) 10%, (D) 20%, (E) 30%, and (F) 40% removal criteria. Node removals within 40% were the most feasible for network construction. (TIF) [file pone.0107925.s003.tif]
