# Supplementary material for: Anomalies in Network Bridges Involved in Bile Acid Metabolism Predict Outcomes of Colorectal Cancer Patients
Source: PLoS One. 2014 Sep 26;9(9):e107925. doi: 10.1371/journal.pone.0107925 (PMC4178056; doi:10.1371/journal.pone.0107925)
Supplement: Table S1 — Top-50 bridge protein information. We showed statistics of each bridge protein about discriminative power (T-score and T-test P) using datasets of Sheffer et al. (DOCX) [file pone.0107925.s005.docx]

**Table S1.** Top-50 bridge protein information. We showed statistics of each bridge protein about discriminative power (T-score and T-test P) using datasets of Sheffer et al.

| Rank | Official gene name | T-score | T-test *P* | Entrez ID | Description |
| --- | --- | --- | --- | --- | --- |
| 1 | PPARGC1A | -12.73 | 4.58E-20 | 10891 | peroxisome proliferator-activated receptor gamma, coactivator 1 alpha |
| 2 | HNF4A | 2.88 | 4.76E-03 | 3172 | hepatocyte nuclear factor 4, alpha |
| 3 | GSK3B | 9.56 | 2.41E-17 | 2932 | glycogen synthase kinase 3 beta |
| 4 | RXRG | -7.01 | 1.47E-10 | 6258 | retinoid X receptor, gamma |
| 5 | CASP8 | 5.62 | 2.17E-07 | 841 | caspase 8, apoptosis-related cysteine peptidase |
| 6 | CREBBP | -1.25 | 2.13E-01 | 1387 | CREB binding protein |
| 7 | PPARA | -0.68 | 5.00E-01 | 5465 | peroxisome proliferator-activated receptor alpha |
| 8 | TP53 | 10.60 | 9.86E-22 | 7157 | tumor protein p53 |
| 9 | EP300 | -3.88 | 2.17E-04 | 2033 | E1A binding protein p300 |
| 10 | RXRA | -8.65 | 1.63E-14 | 6256 | retinoid X receptor, alpha |
| 11 | GAPDH | 3.95 | 1.21E-04 | 2597 | glyceraldehyde-3-phosphate dehydrogenase |
| 12 | PIAS1 | -10.06 | 7.05E-15 | 8554 | protein inhibitor of activated STAT, 1 |
| 13 | SP3 | 4.34 | 3.36E-05 | 6670 | Sp3 transcription factor |
| 14 | RELA | 8.05 | 3.74E-13 | 5970 | v-rel reticuloendotheliosis viral oncogene homolog A (avian) |
| 15 | JUN | 2.69 | 8.60E-03 | 3725 | jun proto-oncogene |
| 16 | THRB | -0.86 | 3.92E-01 | 7068 | thyroid hormone receptor, beta |
| 17 | DDX5 | -2.91 | 4.59E-03 | 1655 | DEAD (Asp-Glu-Ala-Asp) box helicase 5 |
| 18 | IRF2 | -2.86 | 5.37E-03 | 3660 | interferon regulatory factor 2 |
| 19 | CTBP1 | 2.79 | 6.23E-03 | 1487 | C-terminal binding protein 1 |
| 20 | UBE2I | 14.37 | 2.25E-31 | 7329 | ubiquitin-conjugating enzyme E2I |
| 21 | FOS | -4.59 | 1.75E-05 | 2353 | FBJ murine osteosarcoma viral oncogene homolog |
| 22 | HNF1A | 9.81 | 5.73E-18 | 6927 | HNF1 homeobox A |
| 23 | NCL | 11.43 | 2.11E-20 | 4691 | nucleolin |
| 24 | EGR1 | -2.72 | 8.08E-03 | 1958 | early growth response 1 |
| 25 | BCL3 | -1.56 | 1.23E-01 | 602 | B-cell CLL/lymphoma 3 |
| 26 | MYBBP1A | 6.33 | 3.92E-09 | 10514 | MYB binding protein (P160) 1a |
| 27 | GABPA | 2.72 | 7.57E-03 | 2551 | GA binding protein transcription factor, alpha subunit 60kDa |
| 28 | TGS1 | 14.52 | 3.28E-34 | 96764 | trimethylguanosine synthase 1 |
| 29 | EEF1D | 11.08 | 3.34E-22 | 1936 | eukaryotic translation elongation factor 1 delta (guanine nucleotide exchange protein) |
| 30 | PTMA | 9.62 | 1.44E-16 | 5757 | prothymosin, alpha |
| 31 | SUMO4 | 6.67 | 1.46E-09 | 387082 | SMT3 suppressor of mif two 3 homolog 4 (S. cerevisiae) |
| 32 | PPARG | -3.36 | 1.17E-03 | 5468 | peroxisome proliferator-activated receptor gamma |
| 33 | SLC2A4 | -2.04 | 4.41E-02 | 6517 | solute carrier family 2 (facilitated glucose transporter), member 4 |
| 34 | ARNT | -4.30 | 4.43E-05 | 405 | aryl hydrocarbon receptor nuclear translocator |
| 35 | POU2F1 | 5.44 | 5.11E-07 | 5451 | POU class 2 homeobox 1 |
| 36 | HNRNPA3 | 0.24 | 8.08E-01 | 220988 | heterogeneous nuclear ribonucleoprotein A3 |
| 37 | SRF | -3.16 | 2.47E-03 | 6722 | serum response factor (c-fos serum response element-binding transcription factor) |
| 38 | TCF12 | 4.78 | 3.30E-06 | 6938 | transcription factor 12 |
| 39 | LDHA | 6.08 | 1.98E-08 | 3939 | lactate dehydrogenase A |
| 40 | ATF4 | 0.11 | 9.17E-01 | 468 | activating transcription factor 4 (tax-responsive enhancer element B67) |
| 41 | NR3C1 | -11.57 | 4.09E-19 | 2908 | nuclear receptor subfamily 3, group C, member 1 (glucocorticoid receptor) |
| 42 | STK11 | 7.02 | 5.45E-11 | 6794 | serine/threonine kinase 11 |
| 43 | HNRNPU | 10.18 | 2.42E-17 | 3192 | heterogeneous nuclear ribonucleoprotein U (scaffold attachment factor A) |
| 44 | DCTN2 | -9.36 | 9.34E-15 | 10540 | dynactin 2 (p50) |
| 45 | TOP2B | -3.17 | 2.01E-03 | 7155 | topoisomerase (DNA) II beta 180kDa |
| 46 | NACA | 4.14 | 5.61E-05 | 4666 | nascent polypeptide-associated complex alpha subunit |
| 47 | CTNNB1 | 10.88 | 9.68E-21 | 1499 | catenin (cadherin-associated protein), beta 1, 88kDa |
| 48 | MYOD1 | 5.07 | 1.05E-06 | 4654 | myogenic differentiation 1 |
| 49 | CEBPD | -5.89 | 1.85E-07 | 1052 | CCAAT/enhancer binding protein (C/EBP), delta |
| 50 | HDAC1 | -2.57 | 1.19E-02 | 3065 | histone deacetylase 1 |
