# Supplementary material for: Anomalies in Network Bridges Involved in Bile Acid Metabolism Predict Outcomes of Colorectal Cancer Patients
Source: PLoS One. 2014 Sep 26;9(9):e107925. doi: 10.1371/journal.pone.0107925 (PMC4178056; doi:10.1371/journal.pone.0107925)
Supplement: Table S2 — Evidence of bridge proteins involved in the regulation of bile acid homeostasis. Shown was previous literature that identified bridge proteins as being involved in the regulation of bile acid homeostasis. In the second column, we provided literature with definitive evidence that defects of some bridge proteins cause abnormal changes of bile acid levels. In the third and fourth columns, we provided literature with indirect evidence: studies in the third column showing that bridge proteins were regulated by or co-activated with a bile acid sensor; studies in the fourth column showing that bridge proteins regulated enzymes in bile acid metabolism. (DOCX) [file pone.0107925.s006.docx]

**Table S2.** Evidence of bridge proteins involved in the regulation of bile acid homeostasis

| Bridge proteins | Evidence for regulating BA homeostasis* | Evidence of bridge proteins regulated by bile acid sensor** | Evidence of bridge proteins regulating bile acid enzymes*** |
| --- | --- | --- | --- |
| PPARGC1A |  | [1,2] | [3–8] |
| HNF4A | [9,10] | [11] | [4,11–18] |
| PPARA | [19] | [20,21] | [15,19,22,23] |
| TP53 | [24] |  |  |
| EP300 |  | [25] |  |
| RXRA |  | [26] | [15,27,28] |
| JUN |  | [29] | [6,30] |
| FOS |  |  | [31] |
| HNF1A | [32] | [33] | [32] |
| EGR1 |  | [34] |  |
| SLC2A4 |  | [35] |  |
| NR3C1 | [36] | [37] | [38,39] |
| STK11 | [40] | [41] | [40,42] |
| CTNNB1 | [43] | [44] |  |

* Literature in which defects of bridge proteins cause abnormal changes of bile acid levels

** Literature in which bridge proteins are co-activated with or regulated by a bile acid sensor, FXR

*** Literature in which bridge proteins regulate the activity or abundance of bile acid enzymes

**References**

1. Yamagata K, Yoshimochi K, Daitoku H, Hirota K, Fukamizu A (2007) Bile acid represses the peroxisome proliferator-activated receptor-gamma coactivator-1 promoter activity in a small heterodimer partner-dependent manner. Int J Mol Med 19: 751–756.

2. Savkur RS, Thomas JS, Bramlett KS, Gao Y, Michael LF, et al. (2005) Ligand-dependent coactivation of the human bile acid receptor FXR by the peroxisome proliferator-activated receptor gamma coactivator-1alpha. J Pharmacol Exp Ther 312: 170–178.

3. Ponugoti B, Fang S, Kemper JK (2007) Functional interaction of hepatic nuclear factor-4 and peroxisome proliferator-activated receptor-gamma coactivator 1alpha in CYP7A1 regulation is inhibited by a key lipogenic activator, sterol regulatory element-binding protein-1c. Mol Endocrinol 21: 2698–2712.

4. Song K-H, Li T, Chiang JYL (2006) A Prospero-related homeodomain protein is a novel co-regulator of hepatocyte nuclear factor 4alpha that regulates the cholesterol 7alpha-hydroxylase gene. J Biol Chem 281: 10081–10088.

5. Li T, Chiang JYL (2005) Mechanism of rifampicin and pregnane X receptor inhibition of human cholesterol 7 alpha-hydroxylase gene transcription. Am J Physiol Gastrointest Liver Physiol 288: G74–84.

6. Li T, Jahan A, Chiang JYL (2006) Bile acids and cytokines inhibit the human cholesterol 7 alpha-hydroxylase gene via the JNK/c-jun pathway in human liver cells. Hepatology 43: 1202–1210.

7. Shin D-J, Osborne TF (2008) Peroxisome proliferator-activated receptor-gamma coactivator-1alpha activation of CYP7A1 during food restriction and diabetes is still inhibited by small heterodimer partner. J Biol Chem 283: 15089–15096.

8. Shin D-J, Campos J a, Gil G, Osborne TF (2003) PGC-1alpha activates CYP7A1 and bile acid biosynthesis. J Biol Chem 278: 50047–50052.

9. Inoue Y, Yu A-M, Inoue J, Gonzalez FJ (2004) Hepatocyte nuclear factor 4alpha is a central regulator of bile acid conjugation. J Biol Chem 279: 2480–2489.

10. Inoue Y, Yu A-M, Yim SH, Ma X, Krausz KW, et al. (2006) Regulation of bile acid biosynthesis by hepatocyte nuclear factor 4alpha. J Lipid Res 47: 215–227.

11. Sanyal S, Båvner A, Haroniti A, Nilsson L-M, Lundåsen T, et al. (2007) Involvement of corepressor complex subunit GPS2 in transcriptional pathways governing human bile acid biosynthesis. Proc Natl Acad Sci U S A 104: 15665–15670.

12. Li T, Ma H, Chiang JYL (2008) TGFbeta1, TNFalpha, and insulin signaling crosstalk in regulation of the rat cholesterol 7alpha-hydroxylase gene expression. J Lipid Res 49: 1981–1989.

13. Miao J, Fang S, Bae Y, Kemper JK (2006) Functional inhibitory cross-talk between constitutive androstane receptor and hepatic nuclear factor-4 in hepatic lipid/glucose metabolism is mediated by competition for binding to the DR1 motif and to the common coactivators, GRIP-1 and PGC-1alpha. J Biol Chem 281: 14537–14546.

14. Honda A, Salen G, Matsuzaki Y, Batta AK, Xu G, et al. (2005) Disrupted coordinate regulation of farnesoid X receptor target genes in a patient with cerebrotendinous xanthomatosis. J Lipid Res 46: 287–296.

15. Marrapodi M, Chiang JY (2000) Peroxisome proliferator-activated receptor alpha (PPARalpha) and agonist inhibit cholesterol 7alpha-hydroxylase gene (CYP7A1) transcription. J Lipid Res 41: 514–520.

16. Stroup D, Chiang JY (2000) HNF4 and COUP-TFII interact to modulate transcription of the cholesterol 7alpha-hydroxylase gene (CYP7A1). J Lipid Res 41: 1–11.

17. Fang H, Strom SC, Ellis E, Duanmu Z, Fu J, et al. (2007) Positive and negative regulation of human hepatic hydroxysteroid sulfotransferase (SULT2A1) gene transcription by rifampicin: roles of hepatocyte nuclear factor 4alpha and pregnane X receptor. J Pharmacol Exp Ther 323: 586–598.

18. Kamiyama Y, Matsubara T, Yoshinari K, Nagata K, Kamimura H, et al. (2007) Role of human hepatocyte nuclear factor 4alpha in the expression of drug-metabolizing enzymes and transporters in human hepatocytes assessed by use of small interfering RNA. Drug Metab Pharmacokinet 22: 287–298.

19. Li F, Patterson AD, Krausz KW, Tanaka N, Gonzalez FJ (2012) Metabolomics reveals an essential role for peroxisome proliferator-activated receptor α in bile acid homeostasis. J Lipid Res 53: 1625–1635.

20. Stayrook KR, Bramlett KS, Savkur RS, Ficorilli J, Cook T, et al. (2005) Regulation of carbohydrate metabolism by the farnesoid X receptor. Endocrinology 146: 984–991.

21. Byun HW, Hong EM, Park SH, Koh DH, Choi MH, et al. (2014) Pravastatin activates the expression of farnesoid X receptor and liver X receptor alpha in Hep3B cells. Hepatobiliary Pancreat Dis Int 13: 65–73.

22. Fang H, Strom SC, Cai H, Falany CN, Kocarek TA, et al. (2005) Regulation of human hepatic hydroxysteroid sulfotransferase gene expression by the peroxisome proliferator-activated receptor alpha transcription factor. Mol Pharmacol 67: 1257–1267.

23. Jung D, Fried M, Kullak-Ublick G a (2002) Human apical sodium-dependent bile salt transporter gene (SLC10A2) is regulated by the peroxisome proliferator-activated receptor alpha. J Biol Chem 277: 30559–30566.

24. Kim D-H, Lee JW (2011) Tumor suppressor p53 regulates bile acid homeostasis via small heterodimer partner. Proc Natl Acad Sci U S A 108: 12266–12270.

25. Fang S, Tsang S, Jones R, Ponugoti B, Yoon H, et al. (2008) The p300 acetylase is critical for ligand-activated farnesoid X receptor (FXR) induction of SHP. J Biol Chem 283: 35086–35095.

26. Repa JJ, Mangelsdorf DJ (1999) Nuclear receptor regulation of cholesterol and bile acid metabolism. Curr Opin Biotechnol 10: 557–563.

27. Goodwin B, Jones SA, Price RR, Watson MA, McKee DD, et al. (2000) A regulatory cascade of the nuclear receptors FXR, SHP-1, and LRH-1 represses bile acid biosynthesis. Mol Cell 6: 517–526.

28. Okuwaki M, Takada T, Iwayanagi Y, Koh S, Kariya Y, et al. (2007) LXR alpha transactivates mouse organic solute transporter alpha and beta via IR-1 elements shared with FXR. Pharm Res 24: 390–398.

29. He F, Li J, Mu Y, Kuruba R, Ma Z, et al. (2006) Downregulation of endothelin-1 by farnesoid X receptor in vascular endothelial cells. Circ Res 98: 192–199.

30. Gupta S, Stravitz RT, Dent P, Hylemon PB (2001) Down-regulation of cholesterol 7alpha-hydroxylase (CYP7A1) gene expression by bile acids in primary rat hepatocytes is mediated by the c-Jun N-terminal kinase pathway. J Biol Chem 276: 15816–15822.

31. Neimark E, Chen F, Li X, Magid MS, Alasio TM, et al. (2006) c-Fos is a critical mediator of inflammatory-mediated repression of the apical sodium-dependent bile acid transporter. Gastroenterology 131: 554–567.

32. Shih DQ, Bussen M, Sehayek E, Ananthanarayanan M, Shneider BL, et al. (2001) Hepatocyte nuclear factor-1alpha is an essential regulator of bile acid and plasma cholesterol metabolism. Nat Genet 27: 375–382.

33. Zollner G, Wagner M, Fickert P, Geier A, Fuchsbichler A, et al. (2005) Role of nuclear receptors and hepatocyte-enriched transcription factors for Ntcp repression in biliary obstruction in mouse liver. Am J Physiol Gastrointest Liver Physiol 289: G798–805.

34. Allen K, Kim ND, Moon J-O, Copple BL (2010) Upregulation of early growth response factor-1 by bile acids requires mitogen-activated protein kinase signaling. Toxicol Appl Pharmacol 243: 63–67.

35. Shen H, Zhang Y, Ding H, Wang X, Chen L, et al. (2008) Farnesoid X receptor induces GLUT4 expression through FXR response element in the GLUT4 promoter. Cell Physiol Biochem 22: 1–14.

36. Rose AJ, Berriel Díaz M, Reimann A, Klement J, Walcher T, et al. (2011) Molecular control of systemic bile acid homeostasis by the liver glucocorticoid receptor. Cell Metab 14: 123–130.

37. Renga B, D’Amore C, Cipriani S, Mencarelli A, Carino A, et al. (2013) FXR mediates a chromatin looping in the GR promoter thus promoting the resolution of colitis in rodents. Pharmacol Res 77: 1–10.

38. Jung D, Fantin a C, Scheurer U, Fried M, Kullak-Ublick G a (2004) Human ileal bile acid transporter gene ASBT (SLC10A2) is transactivated by the glucocorticoid receptor. Gut 53: 78–84.

39. Fang H, Abdolalipour M, Duanmu Z, Smigelski JR, Weckle A, et al. (2005) Regulation of glucocorticoid-inducible hydroxysteroid sulfotransferase (SULT2A-40/41) gene transcription in primary cultured rat hepatocytes: role of CCAAT/enhancer-binding protein liver-enriched transcription factors. Drug Metab Dispos 33: 147–156.

40. Woods A, Heslegrave AJ, Muckett PJ, Levene AP, Clements M, et al. (2011) LKB1 is required for hepatic bile acid transport and canalicular membrane integrity in mice. Biochem J 434: 49–60.

41. Lee CG, Kim YW, Kim EH, Meng Z, Huang W, et al. (2012) Farnesoid X receptor protects hepatocytes from injury by repressing miR-199a-3p, which increases levels of LKB1. Gastroenterology 142: 1206–1217.e7.

42. Homolya L, Fu D, Sengupta P, Jarnik M, Gillet J-P, et al. (2014) LKB1/AMPK and PKA control ABCB11 trafficking and polarization in hepatocytes. PLoS One 9: e91921.

43. Behari J, Yeh T-H, Krauland L, Otruba W, Cieply B, et al. (2010) Liver-specific beta-catenin knockout mice exhibit defective bile acid and cholesterol homeostasis and increased susceptibility to diet-induced steatohepatitis. Am J Pathol 176: 744–753.

44. Kim I, Morimura K, Shah Y, Yang Q, Ward JM, et al. (2007) Spontaneous hepatocarcinogenesis in farnesoid X receptor-null mice. Carcinogenesis 28: 940–946.
