# Supplementary material for: Anomalies in Network Bridges Involved in Bile Acid Metabolism Predict Outcomes of Colorectal Cancer Patients
Source: PLoS One. 2014 Sep 26;9(9):e107925. doi: 10.1371/journal.pone.0107925 (PMC4178056; doi:10.1371/journal.pone.0107925)
Supplement: Table S3 — Sensor and enzyme proteins in bile acid or glucose metabolism. (DOCX) [file pone.0107925.s007.docx]

**Table S3.** Sensor and enzyme proteins in bile acid or glucose metabolism

| Official  gene name | Metabolism | Type | Entrez ID | Description |
| --- | --- | --- | --- | --- |
| NR1H4 | Bile acid metabolism | sensor | 9971 | nuclear receptor subfamily 1, group H, member 4 |
| SOAT2 | Bile acid metabolism | enzyme | 8435 | sterol O-acyltransferase 2 |
| SOAT1 | Bile acid metabolism | enzyme | 6646 | sterol O-acyltransferase 1 |
| CYP7A1 | Bile acid metabolism | enzyme | 1581 | cytochrome P450, family 7, subfamily A, polypeptide 1 |
| SLC27A5 | Bile acid metabolism | enzyme | 10998 | solute carrier family 27 (fatty acid transporter), member 5 |
| BAAT | Bile acid metabolism | enzyme | 570 | bile acid CoA: amino acid N-acyltransferase (glycine N-choloyltransferase) |
| ACAA1 | Bile acid metabolism | enzyme | 30 | acetyl-CoA acyltransferase 1 |
| SCP2 | Bile acid metabolism | enzyme | 6342 | sterol carrier protein 2 |
| ALDH9A1 | Bile acid metabolism | enzyme | 223 | aldehyde dehydrogenase 9 family, member A1 |
| ALDH3A1 | Bile acid metabolism | enzyme | 218 | aldehyde dehydrogenase 3 family, member A1 |
| ALDH2 | Bile acid metabolism | enzyme | 217 | aldehyde dehydrogenase 2 family (mitochondrial) |
| ALDH1B1 | Bile acid metabolism | enzyme | 219 | aldehyde dehydrogenase 1 family, member B1 |
| ALDH7A1 | Bile acid metabolism | enzyme | 501 | aldehyde dehydrogenase 7 family, member A1 |
| ALDH3A2 | Bile acid metabolism | enzyme | 224 | aldehyde dehydrogenase 3 family, member A2 |
| SLC27A2 | Bile acid metabolism | enzyme | 11001 | solute carrier family 27 (fatty acid transporter), member 2 |
| ADHFE1 | Bile acid metabolism | enzyme | 137872 | alcohol dehydrogenase, iron containing, 1 |
| CYP27A1 | Bile acid metabolism | enzyme | 1593 | cytochrome P450, family 27, subfamily A, polypeptide 1 |
| AKR1D1 | Bile acid metabolism | enzyme | 6718 | aldo-keto reductase family 1, member D1 |
| SRD5A1 | Bile acid metabolism | enzyme | 6715 | steroid-5-alpha-reductase, alpha polypeptide 1 (3-oxo-5 alpha-steroid delta 4-dehydrogenase alpha 1) |
| SRD5A2 | Bile acid metabolism | enzyme | 6716 | steroid-5-alpha-reductase, alpha polypeptide 2 (3-oxo-5 alpha-steroid delta 4-dehydrogenase alpha 2) |
| SRD5A3 | Bile acid metabolism | enzyme | 79644 | steroid 5 alpha-reductase 3 |
| HSD3B1 | Bile acid metabolism | enzyme | 3283 | hydroxy-delta-5-steroid dehydrogenase, 3 beta- and steroid delta-isomerase 1 |
| HSD3B2 | Bile acid metabolism | enzyme | 3284 | hydroxy-delta-5-steroid dehydrogenase, 3 beta- and steroid delta-isomerase 2 |
| CYP8B1 | Bile acid metabolism | enzyme | 1582 | cytochrome P450, family 8, subfamily B, polypeptide 1 |
| CYP7B1 | Bile acid metabolism | enzyme | 9420 | cytochrome P450, family 7, subfamily B, polypeptide 1 |
| CYP3A4 | Bile acid metabolism | enzyme | 1576 | cytochrome P450, family 3, subfamily A, polypeptide 4 |
| ACOX3 | Bile acid metabolism | enzyme | 8310 | acyl-CoA oxidase 3, pristanoyl |
| ACOX2 | Bile acid metabolism | enzyme | 8309 | acyl-CoA oxidase 2, branched chain |
| ACOX1 | Bile acid metabolism | enzyme | 51 | acyl-CoA oxidase 1, palmitoyl |
| EHHADH | Bile acid metabolism | enzyme | 1962 | enoyl-CoA, hydratase/3-hydroxyacyl CoA dehydrogenase |
| ACOT6 | Bile acid metabolism | enzyme | 641372 | acyl-CoA thioesterase 6 |
| ACOT1 | Bile acid metabolism | enzyme | 641371 | acyl-CoA thioesterase 1 |
| ACOT2 | Bile acid metabolism | enzyme | 10965 | acyl-CoA thioesterase 2 |
| ACOT7 | Bile acid metabolism | enzyme | 11332 | acyl-CoA thioesterase 7 |
| ACOT8 | Bile acid metabolism | enzyme | 10005 | acyl-CoA thioesterase 8 |
| ACOT4 | Bile acid metabolism | enzyme | 122970 | acyl-CoA thioesterase 4 |
| STARD3 | Bile acid metabolism | enzyme | 10948 | StAR-related lipid transfer (START) domain containing 3 |
| DBI | Bile acid metabolism | enzyme | 1622 | diazepam binding inhibitor (GABA receptor modulator, acyl-CoA binding protein) |
| ABCA1 | Bile acid metabolism | enzyme | 19 | ATP-binding cassette, sub-family A (ABC1), member 1 |
| SLC6A6 | Bile acid metabolism | enzyme | 6533 | solute carrier family 6 (neurotransmitter transporter, taurine), member 6 |
| SLC32A1 | Bile acid metabolism | enzyme | 140679 | solute carrier family 32 (GABA vesicular transporter), member 1 |
| SLC6A14 | Bile acid metabolism | enzyme | 11254 | solute carrier family 6 (amino acid transporter), member 14 |
| SLC38A2 | Bile acid metabolism | enzyme | 54407 | solute carrier family 38, member 2 |
| SLC38A4 | Bile acid metabolism | enzyme | 55089 | solute carrier family 38, member 4 |
| SLC38A1 | Bile acid metabolism | enzyme | 81539 | solute carrier family 38, member 1 |
| SLC10A1 | Bile acid metabolism | enzyme | 6554 | solute carrier family 10 (sodium/bile acid cotransporter family), member 1 |
| SLC10A2 | Bile acid metabolism | enzyme | 6555 | solute carrier family 10 (sodium/bile acid cotransporter family), member 2 |
| SLCO1A2 | Bile acid metabolism | enzyme | 6579 | solute carrier organic anion transporter family, member 1A2 |
| SLCO1B1 | Bile acid metabolism | enzyme | 10599 | solute carrier organic anion transporter family, member 1B1 |
| SLCO4A1 | Bile acid metabolism | enzyme | 28231 | solute carrier organic anion transporter family, member 4A1 |
| SLCO1B3 | Bile acid metabolism | enzyme | 28234 | solute carrier organic anion transporter family, member 1B3 |
| ABCB11 | Bile acid metabolism | enzyme | 8647 | ATP-binding cassette, sub-family B (MDR/TAP), member 11 |
| ABCC3 | Bile acid metabolism | enzyme | 8714 | ATP-binding cassette, sub-family C (CFTR/MRP), member 3 |
| SLC36A1 | Bile acid metabolism | enzyme | 206358 | solute carrier family 36 (proton/amino acid symporter), member 1 |
| EGLN2 | Glucose metabolism (Glycolysis) | sensor | 112398 | egl nine homolog 2 (C. elegans) |
| EGLN1 | Glucose metabolism (Glycolysis) | sensor | 54583 | egl nine homolog 1 (C. elegans) |
| EGLN3 | Glucose metabolism (Glycolysis) | sensor | 112399 | egl nine homolog 3 (C. elegans) |
| HIF1AN | Glucose metabolism (Glycolysis) | sensor | 55662 | hypoxia inducible factor 1, alpha subunit inhibitor |
| PDHA1 | Glucose metabolism (Glycolysis) | enzyme | 5160 | pyruvate dehydrogenase (lipoamide) alpha 1 |
| PDHB | Glucose metabolism (Glycolysis) | enzyme | 5162 | pyruvate dehydrogenase (lipoamide) beta |
| PDHA2 | Glucose metabolism (Glycolysis) | enzyme | 5161 | pyruvate dehydrogenase (lipoamide) alpha 2 |
| PKM | Glucose metabolism (Glycolysis) | enzyme | 5315 | pyruvate kinase, muscle |
| PDHX | Glucose metabolism (Glycolysis) | enzyme | 8050 | pyruvate dehydrogenase complex, component X |
| DLD | Glucose metabolism (Glycolysis) | enzyme | 1738 | dihydrolipoamide dehydrogenase |
| DLAT | Glucose metabolism (Glycolysis) | enzyme | 1737 | dihydrolipoamide S-acetyltransferase |
| ME1 | Glucose metabolism (Glycolysis) | enzyme | 4199 | malic enzyme 1, NADP(+)-dependent, cytosolic |
| ME2 | Glucose metabolism (Glycolysis) | enzyme | 4200 | malic enzyme 2, NAD(+)-dependent, mitochondrial |
| ME3 | Glucose metabolism (Glycolysis) | enzyme | 10873 | malic enzyme 3, NADP(+)-dependent, mitochondrial |
| ACOT12 | Glucose metabolism (Glycolysis) | enzyme | 134526 | acyl-CoA thioesterase 12 |
| ACSS1 | Glucose metabolism (Glycolysis) | enzyme | 84532 | acyl-CoA synthetase short-chain family member 1 |
| PC | Glucose metabolism (Glycolysis) | enzyme | 5091 | pyruvate carboxylase |
| PCK1 | Glucose metabolism (Glycolysis) | enzyme | 5105 | phosphoenolpyruvate carboxykinase 1 (soluble) |
| PCK2 | Glucose metabolism (Glycolysis) | enzyme | 5106 | phosphoenolpyruvate carboxykinase 2 (mitochondrial) |
| ENO2 | Glucose metabolism (Glycolysis) | enzyme | 2026 | enolase 2 (gamma, neuronal) |
| ENO3 | Glucose metabolism (Glycolysis) | enzyme | 2027 | enolase 3 (beta, muscle) |
| ENO1 | Glucose metabolism (Glycolysis) | enzyme | 2023 | enolase 1, (alpha) |
| AKR1A1 | Glucose metabolism (Glycolysis) | enzyme | 10327 | aldo-keto reductase family 1, member A1 (aldehyde reductase) |
| ALDH9A1 | Glucose metabolism (Glycolysis) | enzyme | 223 | aldehyde dehydrogenase 9 family, member A1 |
| ALDH3A1 | Glucose metabolism (Glycolysis) | enzyme | 218 | aldehyde dehydrogenase 3 family, member A1 |
| ALDH2 | Glucose metabolism (Glycolysis) | enzyme | 217 | aldehyde dehydrogenase 2 family (mitochondrial) |
| ALDH1B1 | Glucose metabolism (Glycolysis) | enzyme | 219 | aldehyde dehydrogenase 1 family, member B1 |
| ALDH7A1 | Glucose metabolism (Glycolysis) | enzyme | 501 | aldehyde dehydrogenase 7 family, member A1 |
| CS | Glucose metabolism (Glycolysis) | enzyme | 1431 | citrate synthase |
| ADHFE1 | Glucose metabolism (Glycolysis) | enzyme | 137872 | alcohol dehydrogenase, iron containing, 1 |
| ALDOB | Glucose metabolism (Glycolysis) | enzyme | 229 | aldolase B, fructose-bisphosphate |
| PGK2 | Glucose metabolism (Glycolysis) | enzyme | 5232 | phosphoglycerate kinase 2 |
| PGAM1 | Glucose metabolism (Glycolysis) | enzyme | 5223 | phosphoglycerate mutase 1 (brain) |
| HK1 | Glucose metabolism (Glycolysis) | enzyme | 3098 | hexokinase 1 |
| G6PC | Glucose metabolism (Glycolysis) | enzyme | 2538 | glucose-6-phosphatase, catalytic subunit |
| PFKFB1 | Glucose metabolism (Glycolysis) | enzyme | 5207 | 6-phosphofructo-2-kinase/fructose-2,6-biphosphatase 1 |
| GPI | Glucose metabolism (Glycolysis) | enzyme | 2821 | glucose-6-phosphate isomerase |
| PFKM | Glucose metabolism (Glycolysis) | enzyme | 5213 | phosphofructokinase, muscle |
| PFKL | Glucose metabolism (Glycolysis) | enzyme | 5211 | phosphofructokinase, liver |
| PFKP | Glucose metabolism (Glycolysis) | enzyme | 5214 | phosphofructokinase, platelet |
| NUP153 | Glucose metabolism (Glycolysis) | enzyme | 9972 | nucleoporin 153kDa |
| NUP160 | Glucose metabolism (Glycolysis) | enzyme | 23279 | nucleoporin 160kDa |
| NUP98 | Glucose metabolism (Glycolysis) | enzyme | 4928 | nucleoporin 98kDa |
| NUP50 | Glucose metabolism (Glycolysis) | enzyme | 10762 | nucleoporin 50kDa |
| NUP133 | Glucose metabolism (Glycolysis) | enzyme | 55746 | nucleoporin 133kDa |
| NUP210 | Glucose metabolism (Glycolysis) | enzyme | 23225 | nucleoporin 210kDa |
| NUP85 | Glucose metabolism (Glycolysis) | enzyme | 79902 | nucleoporin 85kDa |
| NUP93 | Glucose metabolism (Glycolysis) | enzyme | 9688 | nucleoporin 93kDa |
| NUP188 | Glucose metabolism (Glycolysis) | enzyme | 23511 | nucleoporin 188kDa |
| NUP62CL | Glucose metabolism (Glycolysis) | enzyme | 54830 | nucleoporin 62kDa C-terminal like |
| NUP37 | Glucose metabolism (Glycolysis) | enzyme | 79023 | nucleoporin 37kDa |
| NUP214 | Glucose metabolism (Glycolysis) | enzyme | 8021 | nucleoporin 214kDa |
| NUP88 | Glucose metabolism (Glycolysis) | enzyme | 4927 | nucleoporin 88kDa |
| NUP43 | Glucose metabolism (Glycolysis) | enzyme | 348995 | nucleoporin 43kDa |
| NUP205 | Glucose metabolism (Glycolysis) | enzyme | 23165 | nucleoporin 205kDa |
| NUP210L | Glucose metabolism (Glycolysis) | enzyme | 91181 | nucleoporin 210kDa-like |
| NUP155 | Glucose metabolism (Glycolysis) | enzyme | 9631 | nucleoporin 155kDa |
| LOC100133510 | Glucose metabolism (Glycolysis) | enzyme | 100133510 | similar to nucleoporin |
| NUP107 | Glucose metabolism (Glycolysis) | enzyme | 57122 | nucleoporin 107kDa |
| NUP62 | Glucose metabolism (Glycolysis) | enzyme | 23636 | nucleoporin 62kDa |
| NUP35 | Glucose metabolism (Glycolysis) | enzyme | 129401 | nucleoporin 35kDa |
| NUPL1 | Glucose metabolism (Glycolysis) | enzyme | 9818 | nucleoporin like 1 |
| NUPL2 | Glucose metabolism (Glycolysis) | enzyme | 11097 | nucleoporin like 2 |
| NUP54 | Glucose metabolism (Glycolysis) | enzyme | 53371 | nucleoporin 54kDa |
| LOC112663 | Glucose metabolism (Glycolysis) | enzyme | 112663 | similar to nucleoporin 62kD (H. sapiens) |
| LOC112661 | Glucose metabolism (Glycolysis) | enzyme | 112661 | similar to nucleoporin 62kD (H. sapiens) |
| LOC119786 | Glucose metabolism (Glycolysis) | enzyme | 119786 | similar to nucleoporin 98kD; ADIR2; Nup98-Nup96 precursor (H. sapiens) |
| LOC153165 | Glucose metabolism (Glycolysis) | enzyme | 153165 | similar to nucleoporin 50kD (H. sapiens) |
| LOC143514 | Glucose metabolism (Glycolysis) | enzyme | 143514 | similar to nucleoporin 98kD (H. sapiens) |
| LOC143513 | Glucose metabolism (Glycolysis) | enzyme | 143513 | similar to nucleoporin 98kD (H. sapiens) |
| LOC129192 | Glucose metabolism (Glycolysis) | enzyme | 129192 | similar to nucleoporin 50kD; nuclear pore-associated protein 60L (H. sapiens) |
| LOC138630 | Glucose metabolism (Glycolysis) | enzyme | 138630 | similar to nucleoporin 214kD (CAIN) (H. sapiens) |
| LOC83370 | Glucose metabolism (Glycolysis) | enzyme | 83370 | similar to nucleoporin 50kD; nuclear pore-associated protein 60L (H. sapiens) |
| LOC126445 | Glucose metabolism (Glycolysis) | enzyme | 126445 | similar to nucleoporin-like protein 1 (H. sapiens) |
| LOC92767 | Glucose metabolism (Glycolysis) | enzyme | 92767 | similar to nucleoporin 50kD (H. sapiens) |
| LOC83036 | Glucose metabolism (Glycolysis) | enzyme | 83036 | similar to nucleoporin 50kD; nuclear pore-associated protein 60L (H. sapiens) |
| LOC126126 | Glucose metabolism (Glycolysis) | enzyme | 126126 | similar to nucleoporin 62kD (H. sapiens) |
| LOC94757 | Glucose metabolism (Glycolysis) | enzyme | 94757 | similar to nucleoporin 62kD (H. sapiens) |
| LOC94754 | Glucose metabolism (Glycolysis) | enzyme | 94754 | similar to nucleoporin 62kD (H. sapiens) |
| LOC65834 | Glucose metabolism (Glycolysis) | enzyme | 65834 | similar to nucleoporin 62kD (H. sapiens) |
| LOC63471 | Glucose metabolism (Glycolysis) | enzyme | 63471 | similar to nucleoporin 62kD (H. sapiens) |
| LOC63322 | Glucose metabolism (Glycolysis) | enzyme | 63322 | similar to nucleoporin 98kD (H. sapiens) |
| LOC63321 | Glucose metabolism (Glycolysis) | enzyme | 63321 | similar to nucleoporin 98kD (H. sapiens) |
| LOC154245 | Glucose metabolism (Glycolysis) | enzyme | 154245 | similar to nucleoporin 153kD (H. sapiens) |
| LOC147051 | Glucose metabolism (Glycolysis) | enzyme | 147051 | similar to nucleoporin 88kD (H. sapiens) |
| LOC82143 | Glucose metabolism (Glycolysis) | enzyme | 82143 | similar to nucleoporin 50kD; nuclear pore-associated protein 60L (H. sapiens) |
| LOC91611 | Glucose metabolism (Glycolysis) | enzyme | 91611 | similar to nucleoporin 50kD; nuclear pore-associated protein 60L (H. sapiens) |
| LOC96258 | Glucose metabolism (Glycolysis) | enzyme | 96258 | similar to nucleoporin 50kD; nuclear pore-associated protein 60L (H. sapiens) |
| RANBP2 | Glucose metabolism (Glycolysis) | enzyme | 5903 | RAN binding protein 2 |
| SLC25A1 | Glucose metabolism (Glycolysis) | enzyme | 6576 | solute carrier family 25 (mitochondrial carrier; citrate transporter), member 1 |
| SLC25A10 | Glucose metabolism (Glycolysis) | enzyme | 1468 | solute carrier family 25 (mitochondrial carrier; dicarboxylate transporter), member 10 |
| SLC16A1 | Glucose metabolism (Glycolysis) | enzyme | 6566 | solute carrier family 16, member 1 (monocarboxylic acid transporter 1) |
| SLC16A3 | Glucose metabolism (Glycolysis) | enzyme | 9123 | solute carrier family 16, member 3 (monocarboxylic acid transporter 4) |
| SLC16A7 | Glucose metabolism (Glycolysis) | enzyme | 9194 | solute carrier family 16, member 7 (monocarboxylic acid transporter 2) |
| SLC16A8 | Glucose metabolism (Glycolysis) | enzyme | 23539 | solute carrier family 16, member 8 (monocarboxylic acid transporter 3) |
