# Supplementary material for: Anomalies in Network Bridges Involved in Bile Acid Metabolism Predict Outcomes of Colorectal Cancer Patients
Source: PLoS One. 2014 Sep 26;9(9):e107925. doi: 10.1371/journal.pone.0107925 (PMC4178056; doi:10.1371/journal.pone.0107925)
Supplement: Table S4 — Enriched GO terms under corrected p-value<0.01. (DOCX) [file pone.0107925.s008.docx]

**Table S4.** Enriched GO terms under corrected p-value < 0.01

| GO-ID | Category | Corrected p-value | Description |
| --- | --- | --- | --- |
| 6357 | Biological process | 3.25E-15 | regulation of transcription from RNA polymerase II promoter |
| 45944 | Biological process | 3.25E-15 | positive regulation of transcription from RNA polymerase II promoter |
| 45941 | Biological process | 4.77E-15 | positive regulation of transcription |
| 45893 | Biological process | 6.02E-15 | positive regulation of transcription, DNA-dependent |
| 51254 | Biological process | 6.02E-15 | positive regulation of RNA metabolic process |
| 10628 | Biological process | 6.02E-15 | positive regulation of gene expression |
| 45935 | Biological process | 2.64E-14 | positive regulation of nucleobase, nucleoside, nucleotide and nucleic acid metabolic process |
| 10557 | Biological process | 3.78E-14 | positive regulation of macromolecule biosynthetic process |
| 51173 | Biological process | 3.78E-14 | positive regulation of nitrogen compound metabolic process |
| 51252 | Biological process | 5.20E-14 | regulation of RNA metabolic process |
| 31328 | Biological process | 1.01E-13 | positive regulation of cellular biosynthetic process |
| 9891 | Biological process | 1.24E-13 | positive regulation of biosynthetic process |
| 6355 | Biological process | 2.79E-13 | regulation of transcription, DNA-dependent |
| 10604 | Biological process | 1.29E-11 | positive regulation of macromolecule metabolic process |
| 31325 | Biological process | 1.97E-11 | positive regulation of cellular metabolic process |
| 45449 | Biological process | 2.45E-11 | regulation of transcription |
| 10468 | Biological process | 4.28E-11 | regulation of gene expression |
| 9893 | Biological process | 4.38E-11 | positive regulation of metabolic process |
| 19219 | Biological process | 8.47E-11 | regulation of nucleobase, nucleoside, nucleotide and nucleic acid metabolic process |
| 51171 | Biological process | 1.02E-10 | regulation of nitrogen compound metabolic process |
| 10556 | Biological process | 2.17E-10 | regulation of macromolecule biosynthetic process |
| 80090 | Biological process | 6.94E-10 | regulation of primary metabolic process |
| 31326 | Biological process | 6.94E-10 | regulation of cellular biosynthetic process |
| 32583 | Biological process | 6.94E-10 | regulation of gene-specific transcription |
| 9889 | Biological process | 8.04E-10 | regulation of biosynthetic process |
| 60255 | Biological process | 1.35E-09 | regulation of macromolecule metabolic process |
| 16481 | Biological process | 2.11E-09 | negative regulation of transcription |
| 31323 | Biological process | 2.27E-09 | regulation of cellular metabolic process |
| 43193 | Biological process | 6.45E-09 | positive regulation of gene-specific transcription |
| 31324 | Biological process | 7.45E-09 | negative regulation of cellular metabolic process |
| 19222 | Biological process | 7.55E-09 | regulation of metabolic process |
| 10629 | Biological process | 7.55E-09 | negative regulation of gene expression |
| 45934 | Biological process | 8.25E-09 | negative regulation of nucleobase, nucleoside, nucleotide and nucleic acid metabolic process |
| 51172 | Biological process | 9.22E-09 | negative regulation of nitrogen compound metabolic process |
| 10558 | Biological process | 1.81E-08 | negative regulation of macromolecule biosynthetic process |
| 45892 | Biological process | 2.43E-08 | negative regulation of transcription, DNA-dependent |
| 9892 | Biological process | 2.43E-08 | negative regulation of metabolic process |
| 31327 | Biological process | 2.46E-08 | negative regulation of cellular biosynthetic process |
| 51253 | Biological process | 2.82E-08 | negative regulation of RNA metabolic process |
| 9890 | Biological process | 2.89E-08 | negative regulation of biosynthetic process |
| 48518 | Biological process | 7.43E-08 | positive regulation of biological process |
| 10605 | Biological process | 7.61E-08 | negative regulation of macromolecule metabolic process |
| 48522 | Biological process | 7.61E-08 | positive regulation of cellular process |
| 10551 | Biological process | 3.22E-07 | regulation of gene-specific transcription from RNA polymerase II promoter |
| 10552 | Biological process | 3.87E-07 | positive regulation of gene-specific transcription from RNA polymerase II promoter |
| 1889 | Biological process | 5.98E-07 | liver development |
| 61008 | Biological process | 6.46E-07 | hepaticobiliary system development |
| 48523 | Biological process | 7.26E-07 | negative regulation of cellular process |
| 9991 | Biological process | 1.23E-06 | response to extracellular stimulus |
| 48646 | Biological process | 1.97E-06 | anatomical structure formation involved in morphogenesis |
| 122 | Biological process | 2.35E-06 | negative regulation of transcription from RNA polymerase II promoter |
| 48513 | Biological process | 2.64E-06 | organ development |
| 48519 | Biological process | 3.10E-06 | negative regulation of biological process |
| 14070 | Biological process | 4.76E-06 | response to organic cyclic substance |
| 30154 | Biological process | 5.04E-06 | cell differentiation |
| 50794 | Biological process | 5.72E-06 | regulation of cellular process |
| 48869 | Biological process | 7.35E-06 | cellular developmental process |
| 51094 | Biological process | 1.06E-05 | positive regulation of developmental process |
| 45597 | Biological process | 1.18E-05 | positive regulation of cell differentiation |
| 34097 | Biological process | 1.23E-05 | response to cytokine stimulus |
| 43009 | Biological process | 1.38E-05 | chordate embryonic development |
| 9792 | Biological process | 1.48E-05 | embryonic development ending in birth or egg hatching |
| 65007 | Biological process | 2.07E-05 | biological regulation |
| 50789 | Biological process | 2.21E-05 | regulation of biological process |
| 50793 | Biological process | 2.75E-05 | regulation of developmental process |
| 2521 | Biological process | 3.06E-05 | leukocyte differentiation |
| 1701 | Biological process | 4.69E-05 | in utero embryonic development |
| 48731 | Biological process | 4.79E-05 | system development |
| 30522 | Biological process | 4.92E-05 | intracellular receptor mediated signaling pathway |
| 48545 | Biological process | 5.89E-05 | response to steroid hormone stimulus |
| 7568 | Biological process | 6.15E-05 | aging |
| 9725 | Biological process | 6.18E-05 | response to hormone stimulus |
| 44419 | Biological process | 6.18E-05 | interspecies interaction between organisms |
| 32502 | Biological process | 6.23E-05 | developmental process |
| 10033 | Biological process | 6.37E-05 | response to organic substance |
| 9653 | Biological process | 6.72E-05 | anatomical structure morphogenesis |
| 10035 | Biological process | 7.75E-05 | response to inorganic substance |
| 1890 | Biological process | 7.97E-05 | placenta development |
| 6350 | Biological process | 8.48E-05 | transcription |
| 31667 | Biological process | 8.56E-05 | response to nutrient levels |
| 9790 | Biological process | 9.01E-05 | embryonic development |
| 9888 | Biological process | 9.24E-05 | tissue development |
| 42221 | Biological process | 1.03E-04 | response to chemical stimulus |
| 42493 | Biological process | 1.15E-04 | response to drug |
| 9719 | Biological process | 1.31E-04 | response to endogenous stimulus |
| 51704 | Biological process | 1.36E-04 | multi-organism process |
| 9887 | Biological process | 1.36E-04 | organ morphogenesis |
| 8285 | Biological process | 1.52E-04 | negative regulation of cell proliferation |
| 48856 | Biological process | 1.58E-04 | anatomical structure development |
| 44238 | Biological process | 1.69E-04 | primary metabolic process |
| 60395 | Biological process | 1.74E-04 | SMAD protein signal transduction |
| 6979 | Biological process | 1.87E-04 | response to oxidative stress |
| 51716 | Biological process | 1.89E-04 | cellular response to stimulus |
| 70887 | Biological process | 2.06E-04 | cellular response to chemical stimulus |
| 44260 | Biological process | 2.07E-04 | cellular macromolecule metabolic process |
| 7275 | Biological process | 2.07E-04 | multicellular organismal development |
| 9605 | Biological process | 2.79E-04 | response to external stimulus |
| 51098 | Biological process | 2.81E-04 | regulation of binding |
| 45595 | Biological process | 2.81E-04 | regulation of cell differentiation |
| 30217 | Biological process | 3.81E-04 | T cell differentiation |
| 51239 | Biological process | 3.81E-04 | regulation of multicellular organismal process |
| 45165 | Biological process | 5.36E-04 | cell fate commitment |
| 48598 | Biological process | 5.40E-04 | embryonic morphogenesis |
| 43170 | Biological process | 5.40E-04 | macromolecule metabolic process |
| 32025 | Biological process | 6.07E-04 | response to cobalt ion |
| 30097 | Biological process | 6.12E-04 | hemopoiesis |
| 42592 | Biological process | 6.12E-04 | homeostatic process |
| 44237 | Biological process | 6.29E-04 | cellular metabolic process |
| 9987 | Biological process | 6.32E-04 | cellular process |
| 32501 | Biological process | 6.59E-04 | multicellular organismal process |
| 65008 | Biological process | 6.84E-04 | regulation of biological quality |
| 22008 | Biological process | 7.53E-04 | neurogenesis |
| 9409 | Biological process | 8.02E-04 | response to cold |
| 1525 | Biological process | 8.15E-04 | angiogenesis |
| 30224 | Biological process | 9.22E-04 | monocyte differentiation |
| 51101 | Biological process | 9.34E-04 | regulation of DNA binding |
| 90047 | Biological process | 9.75E-04 | positive regulation of transcription regulator activity |
| 51091 | Biological process | 9.75E-04 | positive regulation of transcription factor activity |
| 48534 | Biological process | 9.82E-04 | hemopoietic or lymphoid organ development |
| 48871 | Biological process | 1.10E-03 | multicellular organismal homeostasis |
| 61061 | Biological process | 1.10E-03 | muscle structure development |
| 302 | Biological process | 1.14E-03 | response to reactive oxygen species |
| 42127 | Biological process | 1.15E-03 | regulation of cell proliferation |
| 46320 | Biological process | 1.21E-03 | regulation of fatty acid oxidation |
| 33235 | Biological process | 1.24E-03 | positive regulation of protein sumoylation |
| 33233 | Biological process | 1.24E-03 | regulation of protein sumoylation |
| 10560 | Biological process | 1.24E-03 | positive regulation of glycoprotein biosynthetic process |
| 7494 | Biological process | 1.24E-03 | midgut development |
| 2520 | Biological process | 1.26E-03 | immune system development |
| 9059 | Biological process | 1.36E-03 | macromolecule biosynthetic process |
| 33993 | Biological process | 1.36E-03 | response to lipid |
| 90304 | Biological process | 1.41E-03 | nucleic acid metabolic process |
| 43388 | Biological process | 1.41E-03 | positive regulation of DNA binding |
| 10467 | Biological process | 1.48E-03 | gene expression |
| 30098 | Biological process | 1.51E-03 | lymphocyte differentiation |
| 8152 | Biological process | 1.52E-03 | metabolic process |
| 60070 | Biological process | 1.54E-03 | canonical Wnt receptor signaling pathway |
| 31668 | Biological process | 1.54E-03 | cellular response to extracellular stimulus |
| 71496 | Biological process | 1.58E-03 | cellular response to external stimulus |
| 6351 | Biological process | 1.58E-03 | transcription, DNA-dependent |
| 2573 | Biological process | 1.61E-03 | myeloid leukocyte differentiation |
| 1824 | Biological process | 1.61E-03 | blastocyst development |
| 32774 | Biological process | 1.67E-03 | RNA biosynthetic process |
| 51147 | Biological process | 1.72E-03 | regulation of muscle cell differentiation |
| 30521 | Biological process | 1.84E-03 | androgen receptor signaling pathway |
| 33273 | Biological process | 1.84E-03 | response to vitamin |
| 60768 | Biological process | 2.00E-03 | regulation of epithelial cell proliferation involved in prostate gland development |
| 50872 | Biological process | 2.00E-03 | white fat cell differentiation |
| 48699 | Biological process | 2.14E-03 | generation of neurons |
| 60249 | Biological process | 2.15E-03 | anatomical structure homeostasis |
| 6950 | Biological process | 2.27E-03 | response to stress |
| 51099 | Biological process | 2.28E-03 | positive regulation of binding |
| 7399 | Biological process | 2.58E-03 | nervous system development |
| 60541 | Biological process | 2.58E-03 | respiratory system development |
| 43627 | Biological process | 2.65E-03 | response to estrogen stimulus |
| 32496 | Biological process | 2.81E-03 | response to lipopolysaccharide |
| 51789 | Biological process | 2.89E-03 | response to protein stimulus |
| 6983 | Biological process | 2.97E-03 | ER overload response |
| 46321 | Biological process | 2.97E-03 | positive regulation of fatty acid oxidation |
| 42110 | Biological process | 3.02E-03 | T cell activation |
| 60429 | Biological process | 3.02E-03 | epithelium development |
| 51591 | Biological process | 3.02E-03 | response to cAMP |
| 51246 | Biological process | 3.07E-03 | regulation of protein metabolic process |
| 8361 | Biological process | 3.09E-03 | regulation of cell size |
| 7517 | Biological process | 3.09E-03 | muscle organ development |
| 34641 | Biological process | 3.09E-03 | cellular nitrogen compound metabolic process |
| 48593 | Biological process | 3.11E-03 | camera-type eye morphogenesis |
| 48514 | Biological process | 3.11E-03 | blood vessel morphogenesis |
| 14706 | Biological process | 3.14E-03 | striated muscle tissue development |
| 7507 | Biological process | 3.14E-03 | heart development |
| 51130 | Biological process | 3.16E-03 | positive regulation of cellular component organization |
| 32868 | Biological process | 3.16E-03 | response to insulin stimulus |
| 33500 | Biological process | 3.16E-03 | carbohydrate homeostasis |
| 42593 | Biological process | 3.16E-03 | glucose homeostasis |
| 51150 | Biological process | 3.28E-03 | regulation of smooth muscle cell differentiation |
| 60742 | Biological process | 3.28E-03 | epithelial cell differentiation involved in prostate gland development |
| 2237 | Biological process | 3.50E-03 | response to molecule of bacterial origin |
| 34599 | Biological process | 3.61E-03 | cellular response to oxidative stress |
| 48565 | Biological process | 3.61E-03 | digestive tract development |
| 6366 | Biological process | 3.61E-03 | transcription from RNA polymerase II promoter |
| 31401 | Biological process | 3.61E-03 | positive regulation of protein modification process |
| 48384 | Biological process | 3.80E-03 | retinoic acid receptor signaling pathway |
| 60537 | Biological process | 3.80E-03 | muscle tissue development |
| 34645 | Biological process | 3.80E-03 | cellular macromolecule biosynthetic process |
| 45444 | Biological process | 3.92E-03 | fat cell differentiation |
| 19216 | Biological process | 3.96E-03 | regulation of lipid metabolic process |
| 31399 | Biological process | 4.04E-03 | regulation of protein modification process |
| 71310 | Biological process | 4.04E-03 | cellular response to organic substance |
| 42594 | Biological process | 4.04E-03 | response to starvation |
| 90046 | Biological process | 4.20E-03 | regulation of transcription regulator activity |
| 51090 | Biological process | 4.20E-03 | regulation of transcription factor activity |
| 45687 | Biological process | 4.20E-03 | positive regulation of glial cell differentiation |
| 10559 | Biological process | 4.20E-03 | regulation of glycoprotein biosynthetic process |
| 31058 | Biological process | 4.20E-03 | positive regulation of histone modification |
| 19217 | Biological process | 4.34E-03 | regulation of fatty acid metabolic process |
| 9612 | Biological process | 4.53E-03 | response to mechanical stimulus |
| 30518 | Biological process | 4.70E-03 | steroid hormone receptor signaling pathway |
| 6807 | Biological process | 4.70E-03 | nitrogen compound metabolic process |
| 32330 | Biological process | 4.70E-03 | regulation of chondrocyte differentiation |
| 45453 | Biological process | 4.70E-03 | bone resorption |
| 14015 | Biological process | 4.70E-03 | positive regulation of gliogenesis |
| 1829 | Biological process | 4.70E-03 | trophectodermal cell differentiation |
| 9749 | Biological process | 4.82E-03 | response to glucose stimulus |
| 6913 | Biological process | 4.97E-03 | nucleocytoplasmic transport |
| 55123 | Biological process | 4.97E-03 | digestive system development |
| 1894 | Biological process | 4.97E-03 | tissue homeostasis |
| 51169 | Biological process | 5.03E-03 | nuclear transport |
| 6006 | Biological process | 5.03E-03 | glucose metabolic process |
| 7519 | Biological process | 5.35E-03 | skeletal muscle tissue development |
| 42542 | Biological process | 5.35E-03 | response to hydrogen peroxide |
| 9746 | Biological process | 5.54E-03 | response to hexose stimulus |
| 34284 | Biological process | 5.54E-03 | response to monosaccharide stimulus |
| 1568 | Biological process | 5.70E-03 | blood vessel development |
| 60538 | Biological process | 5.73E-03 | skeletal muscle organ development |
| 10038 | Biological process | 5.77E-03 | response to metal ion |
| 70542 | Biological process | 5.77E-03 | response to fatty acid |
| 9628 | Biological process | 5.84E-03 | response to abiotic stimulus |
| 33189 | Biological process | 5.87E-03 | response to vitamin A |
| 6139 | Biological process | 6.18E-03 | nucleobase, nucleoside, nucleotide and nucleic acid metabolic process |
| 1944 | Biological process | 6.30E-03 | vasculature development |
| 50769 | Biological process | 6.30E-03 | positive regulation of neurogenesis |
| 61035 | Biological process | 6.33E-03 | regulation of cartilage development |
| 10149 | Biological process | 6.33E-03 | senescence |
| 48729 | Biological process | 6.40E-03 | tissue morphogenesis |
| 16043 | Biological process | 6.98E-03 | cellular component organization |
| 10907 | Biological process | 6.98E-03 | positive regulation of glucose metabolic process |
| 45445 | Biological process | 6.98E-03 | myoblast differentiation |
| 32535 | Biological process | 7.14E-03 | regulation of cellular component size |
| 48592 | Biological process | 7.15E-03 | eye morphogenesis |
| 45637 | Biological process | 7.40E-03 | regulation of myeloid cell differentiation |
| 7584 | Biological process | 7.49E-03 | response to nutrient |
| 30855 | Biological process | 7.49E-03 | epithelial cell differentiation |
| 1569 | Biological process | 7.49E-03 | patterning of blood vessels |
| 48639 | Biological process | 7.49E-03 | positive regulation of developmental growth |
| 1825 | Biological process | 7.49E-03 | blastocyst formation |
| 48469 | Biological process | 7.49E-03 | cell maturation |
| 6996 | Biological process | 7.59E-03 | organelle organization |
| 6066 | Biological process | 7.59E-03 | alcohol metabolic process |
| 32270 | Biological process | 7.59E-03 | positive regulation of cellular protein metabolic process |
| 51170 | Biological process | 7.63E-03 | nuclear import |
| 16070 | Biological process | 7.63E-03 | RNA metabolic process |
| 9743 | Biological process | 7.87E-03 | response to carbohydrate stimulus |
| 43525 | Biological process | 8.04E-03 | positive regulation of neuron apoptosis |
| 46849 | Biological process | 8.04E-03 | bone remodeling |
| 43065 | Biological process | 8.20E-03 | positive regulation of apoptosis |
| 35295 | Biological process | 8.20E-03 | tube development |
| 7369 | Biological process | 8.30E-03 | gastrulation |
| 43068 | Biological process | 8.39E-03 | positive regulation of programmed cell death |
| 10565 | Biological process | 8.52E-03 | regulation of cellular ketone metabolic process |
| 9607 | Biological process | 8.52E-03 | response to biotic stimulus |
| 45913 | Biological process | 8.55E-03 | positive regulation of carbohydrate metabolic process |
| 10676 | Biological process | 8.55E-03 | positive regulation of cellular carbohydrate metabolic process |
| 1836 | Biological process | 8.55E-03 | release of cytochrome c from mitochondria |
| 10942 | Biological process | 8.58E-03 | positive regulation of cell death |
| 33554 | Biological process | 8.93E-03 | cellular response to stress |
| 51247 | Biological process | 9.11E-03 | positive regulation of protein metabolic process |
| 10720 | Biological process | 9.18E-03 | positive regulation of cell development |
| 31056 | Biological process | 9.21E-03 | regulation of histone modification |
| 35466 | Biological process | 9.23E-03 | regulation of signaling pathway |
| 32582 | Biological process | 9.38E-03 | negative regulation of gene-specific transcription |
| 19318 | Biological process | 9.62E-03 | hexose metabolic process |
| 46649 | Biological process | 9.78E-03 | lymphocyte activation |
| 45685 | Biological process | 9.84E-03 | regulation of glial cell differentiation |
| 45923 | Biological process | 9.84E-03 | positive regulation of fatty acid metabolic process |
| 43434 | Biological process | 9.86E-03 | response to peptide hormone stimulus |
| 30528 | Molecular function | 2.77E-16 | transcription regulator activity |
| 8134 | Molecular function | 5.36E-16 | transcription factor binding |
| 3677 | Molecular function | 2.20E-15 | DNA binding |
| 3700 | Molecular function | 6.23E-14 | transcription factor activity |
| 3676 | Molecular function | 9.69E-14 | nucleic acid binding |
| 16563 | Molecular function | 9.97E-14 | transcription activator activity |
| 10843 | Molecular function | 9.97E-14 | promoter binding |
| 43565 | Molecular function | 9.97E-14 | sequence-specific DNA binding |
| 44212 | Molecular function | 1.21E-13 | DNA regulatory region binding |
| 3702 | Molecular function | 2.80E-10 | RNA polymerase II transcription factor activity |
| 3690 | Molecular function | 1.75E-09 | double-stranded DNA binding |
| 43566 | Molecular function | 4.54E-08 | structure-specific DNA binding |
| 3713 | Molecular function | 4.67E-08 | transcription coactivator activity |
| 3712 | Molecular function | 2.85E-07 | transcription cofactor activity |
| 35257 | Molecular function | 6.37E-07 | nuclear hormone receptor binding |
| 5515 | Molecular function | 6.37E-07 | protein binding |
| 46982 | Molecular function | 7.42E-07 | protein heterodimerization activity |
| 51427 | Molecular function | 1.17E-06 | hormone receptor binding |
| 16564 | Molecular function | 1.44E-06 | transcription repressor activity |
| 3707 | Molecular function | 1.46E-06 | steroid hormone receptor activity |
| 46983 | Molecular function | 1.46E-06 | protein dimerization activity |
| 4879 | Molecular function | 1.89E-06 | ligand-dependent nuclear receptor activity |
| 70412 | Molecular function | 2.21E-05 | R-SMAD binding |
| 3704 | Molecular function | 3.25E-05 | specific RNA polymerase II transcription factor activity |
| 35258 | Molecular function | 4.13E-05 | steroid hormone receptor binding |
| 46332 | Molecular function | 4.74E-05 | SMAD binding |
| 51059 | Molecular function | 9.19E-05 | NF-kappaB binding |
| 19900 | Molecular function | 9.81E-05 | kinase binding |
| 4886 | Molecular function | 1.31E-04 | retinoid-X receptor activity |
| 50681 | Molecular function | 4.03E-04 | androgen receptor binding |
| 3682 | Molecular function | 4.86E-04 | chromatin binding |
| 19901 | Molecular function | 5.11E-04 | protein kinase binding |
| 3708 | Molecular function | 5.73E-04 | retinoic acid receptor activity |
| 35326 | Molecular function | 7.07E-04 | enhancer binding |
| 3705 | Molecular function | 7.07E-04 | RNA polymerase II transcription factor activity, enhancer binding |
| 16566 | Molecular function | 7.43E-04 | specific transcriptional repressor activity |
| 19899 | Molecular function | 9.26E-04 | enzyme binding |
| 33613 | Molecular function | 9.26E-04 | transcription activator binding |
| 70491 | Molecular function | 2.49E-03 | transcription repressor binding |
| 8022 | Molecular function | 2.68E-03 | protein C-terminus binding |
| 5496 | Molecular function | 3.11E-03 | steroid binding |
| 5488 | Molecular function | 6.93E-03 | binding |
| 5102 | Molecular function | 9.84E-03 | receptor binding |
| 5634 | Cellular component | 1.20E-12 | nucleus |
| 44451 | Cellular component | 3.21E-10 | nucleoplasm part |
| 31981 | Cellular component | 3.21E-10 | nuclear lumen |
| 5654 | Cellular component | 1.00E-09 | nucleoplasm |
| 70013 | Cellular component | 1.19E-09 | intracellular organelle lumen |
| 5667 | Cellular component | 1.22E-09 | transcription factor complex |
| 43233 | Cellular component | 1.27E-09 | organelle lumen |
| 31974 | Cellular component | 1.66E-09 | membrane-enclosed lumen |
| 44428 | Cellular component | 1.98E-09 | nuclear part |
| 43231 | Cellular component | 1.58E-07 | intracellular membrane-bounded organelle |
| 43227 | Cellular component | 1.58E-07 | membrane-bounded organelle |
| 43229 | Cellular component | 6.23E-07 | intracellular organelle |
| 43226 | Cellular component | 6.23E-07 | organelle |
| 44424 | Cellular component | 8.15E-07 | intracellular part |
| 32991 | Cellular component | 2.08E-06 | macromolecular complex |
| 5622 | Cellular component | 2.64E-06 | intracellular |
| 43234 | Cellular component | 8.83E-06 | protein complex |
| 34747 | Cellular component | 1.73E-04 | Axin-APC-beta-catenin-GSK3B complex |
| 5829 | Cellular component | 5.42E-04 | cytosol |
| 44446 | Cellular component | 2.60E-03 | intracellular organelle part |
| 44422 | Cellular component | 3.10E-03 | organelle part |
| 16604 | Cellular component | 9.83E-03 | nuclear body |
