# Supplementary material for: Anomalies in Network Bridges Involved in Bile Acid Metabolism Predict Outcomes of Colorectal Cancer Patients
Source: PLoS One. 2014 Sep 26;9(9):e107925. doi: 10.1371/journal.pone.0107925 (PMC4178056; doi:10.1371/journal.pone.0107925)
Supplement: Table S5 — Enriched KEGG non-metabolic pathways under FDR-adjusted hypergeometric p-value<0.01. (DOCX) [file pone.0107925.s009.docx]

**Table S5.** Enriched KEGG non-metabolic pathways under FDR-adjusted hypergeometric p-value < 0.01

| KEGG pathway name | KEGG ID | Hypergeometric p-value | FDR adjusted p-value (q-value) |
| --- | --- | --- | --- |
| Pathways in cancer | hsa05200 | 5.78E-12 | 6.94E-10 |
| Adipocytokine signaling pathway | hsa04920 | 2.30E-09 | 1.38E-07 |
| Prostate cancer | hsa05215 | 1.33E-08 | 5.32E-07 |
| Thyroid cancer | hsa05216 | 3.05E-08 | 9.15E-07 |
| Huntington's disease | hsa05016 | 4.92E-07 | 1.18E-05 |
| Colorectal cancer | hsa05210 | 1.40E-06 | 2.80E-05 |
| Wnt signaling pathway | hsa04310 | 2.61E-06 | 4.47E-05 |
| Notch signaling pathway | hsa04330 | 3.12E-06 | 4.59E-05 |
| Small cell lung cancer | hsa05222 | 3.44E-06 | 4.59E-05 |
| B cell receptor signaling pathway | hsa04662 | 3.83E-05 | 4.35E-04 |
| Chronic myeloid leukemia | hsa05220 | 4.60E-05 | 4.35E-04 |
| PPAR signaling pathway | hsa03320 | 4.60E-05 | 4.35E-04 |
| Neurotrophin signaling pathway | hsa04722 | 4.71E-05 | 4.35E-04 |
| Renal cell carcinoma | hsa05211 | 9.88E-05 | 8.47E-04 |
| Endometrial cancer | hsa05213 | 1.59E-04 | 1.27E-03 |
| Cell cycle | hsa04110 | 1.93E-04 | 1.36E-03 |
| Basal cell carcinoma | hsa05217 | 1.95E-04 | 1.36E-03 |
| T cell receptor signaling pathway | hsa04660 | 2.06E-04 | 1.36E-03 |
| Melanogenesis | hsa04916 | 2.15E-04 | 1.36E-03 |
| Toll-like receptor signaling pathway | hsa04620 | 2.33E-04 | 1.40E-03 |
| Chagas disease | hsa05142 | 2.53E-04 | 1.45E-03 |
| Non-small cell lung cancer | hsa05223 | 3.38E-04 | 1.84E-03 |
| Long-term potentiation | hsa04720 | 3.78E-04 | 1.97E-03 |
| MAPK signaling pathway | hsa04010 | 4.58E-04 | 2.29E-03 |
| Leishmaniasis | hsa05140 | 5.44E-04 | 2.61E-03 |
| Maturity onset diabetes of the young | hsa04950 | 6.07E-04 | 2.80E-03 |
| Apoptosis | hsa04210 | 1.74E-03 | 7.72E-03 |
