# Supplementary material for: Anomalies in Network Bridges Involved in Bile Acid Metabolism Predict Outcomes of Colorectal Cancer Patients
Source: PLoS One. 2014 Sep 26;9(9):e107925. doi: 10.1371/journal.pone.0107925 (PMC4178056; doi:10.1371/journal.pone.0107925)
Supplement: Text S1 — Characteristics of bridgeness scores. (DOCX) [file pone.0107925.s012.docx]

Supporting Information Text

## ***Comparison of bridgeness score with existing centralities***

We first assessed the differences between bridgeness score and existing centralities, including those for degree, closeness and betweenness, by calculating Pearson’s correlation coefficients. Before these calculations, we generated a small sample network (**Figure S5A**). This network was designed to be scale-free network to resemble our bridge network (**Figure S6**).

In examining differences between bridgeness and existing centralities, we postulated two situations, one in which sensors and enzymes are node hubs in the network (e.g., nodes-1 and -6 represent a sensor and an enzyme, respectively), and the other in which sensors and enzymes are non-hub nodes in the network (e.g., nodes-2 and -3 represent a sensor and an enzyme, respectively).

Intriguingly, correlations between bridgeness and existing centralities varied according to the postulated situations. When a sensor and an enzyme were hub nodes, Pearson’s correlation coefficients between bridgeness score and existing centralities were likely to be high (*ρ* > 0.86; **Figure S5B**). However, when a sensor and an enzyme were non-hub nodes, Pearson’s correlation coefficients between bridgeness score and existing centralities were likely to be low (*ρ* < 0.69; **Figure S5B**). Surprisingly, a sensor and enzymes in a BA-bridge network did not show high hubness; their degrees were lower than the average degree of overall nodes; the degrees of a sensor, enzymes, and overall nodes were 13, 5.91, and 15.26, respectively. Therefore, we concluded that bridgeness score is likely to differ from existing centralities in our bridge network.

## ***Ability to examine proteins within paths between sensors and enzymes***

We tested whether bridgeness score could identify proteins that are densely connected within paths between sensors and enzymes. Bridgeness score was designed to examine a particular protein’s connections within specific paths between sensors and enzymes, regardless of the connections within other unrelated paths. In contrast, existing centralities incorporate other unrelated paths into their calculations. Therefore, we expected that bridgeness score would likely identify proteins located within paths between sensors and enzymes.

Interestingly, the top-ranked proteins selected by bridgeness score were included in the shortest paths between sensors and enzymes (**Table S6**). For example, the highest bridgeness protein, PPARGC1A, was located within the shortest paths between a BA sensor and BA enzymes. Additionally, the number of shortest paths passing PPARGC1A was the most (*N* = 10) among proteins in the network. However, other existing centralities underrated PPARGC1A in their calculations; ranks of PPARGC1A by existing centralities were lower than 479 (degree rank = 479.5; closeness rank = 733.5, betweenness rank = 1134). This finding suggested that bridgeness could identify proteins located within paths between sensors and enzymes, while other existing centralities could not.

Also, using bridgeness, we could predict proteins on the shortest paths between a sensor and enzymes in a BA-bridge network (**Figure S1**). Using receiver operating characteristic (ROC) curves and areas under the curve (AUCs), we measured the accuracy of predictions about proteins on the shortest paths (See the 31 proteins on the shortest paths in **Table S7**). The AUC was higher for bridgeness than for existing centralities (**Figure S7B**), emphasizing that proteins selected by high bridgeness were likely to be located within paths between sensors and enzymes. Hence, we utilized bridgeness score to identify proteins that are densely connected within paths between sensors and enzymes.

**Supporting Information Figures**

**
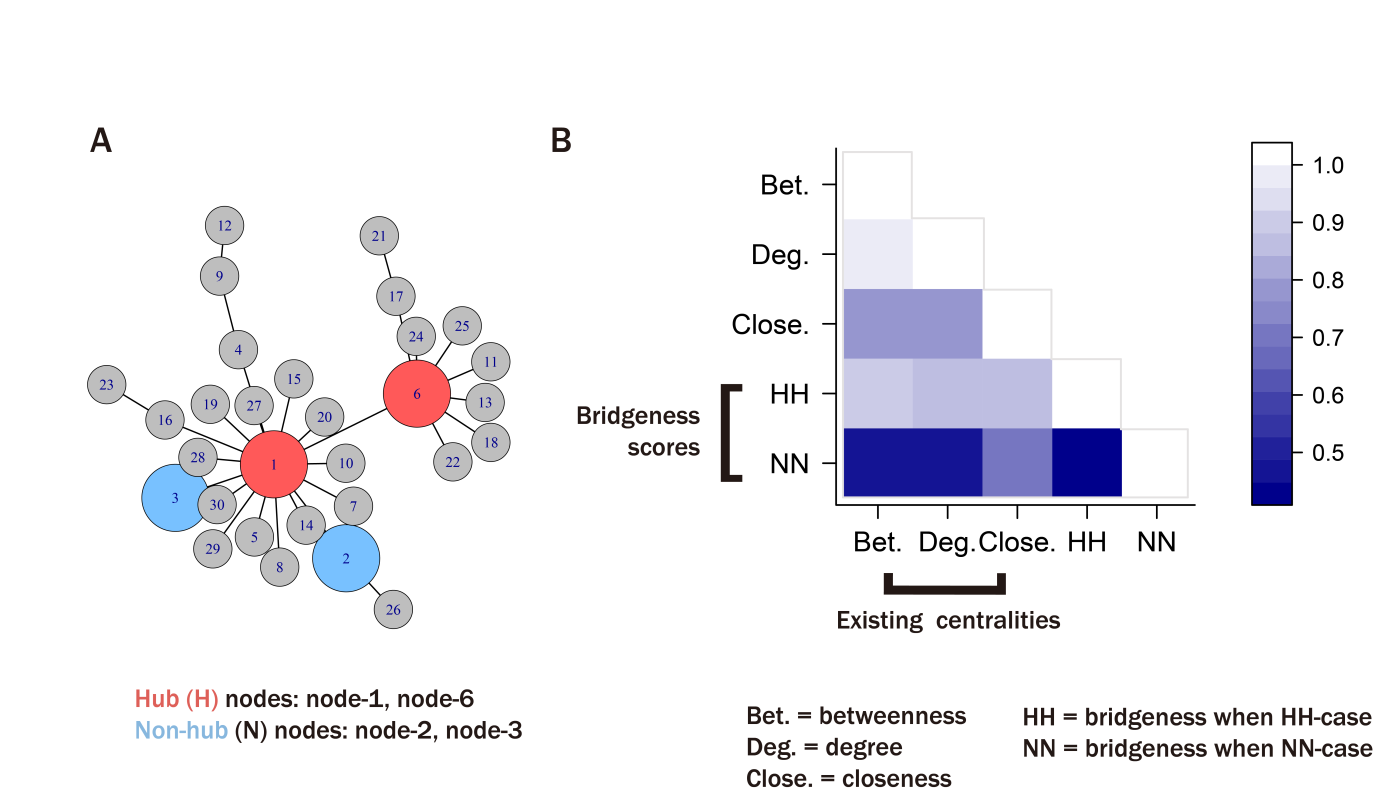
**

**Figure S5. Comparisons of bridgeness score with existing centralities in a sample network.** (**A**) Sample network with 30 nodes. Nodes-1 and -6 (red) are hub nodes in the network and represent a situation in which a sensor and an enzyme are hub nodes. Nodes-2 and -3 (blue) are non-hub nodes and represent a situation in which a sensor and an enzyme are non-hub nodes. (**B**) Pairwise comparisons of applied scores by Pearson’s correlation coefficients. The applied scores were betweenness, degree, closeness, and bridgeness when a sensor and an enzyme were hub-nodes (HH-case), and bridgeness when a sensor and an enzyme were non-hub nodes (NN-case). Each cell indicates Pearson’s correlation coefficient between a score denoted in a row and a score denoted in a column. If the color of a cell became whiter, then the correlation between scores denoted in a row and a column became higher; if the color of a cell became bluer, then the correlation between scores denoted in a row and a column became lower.


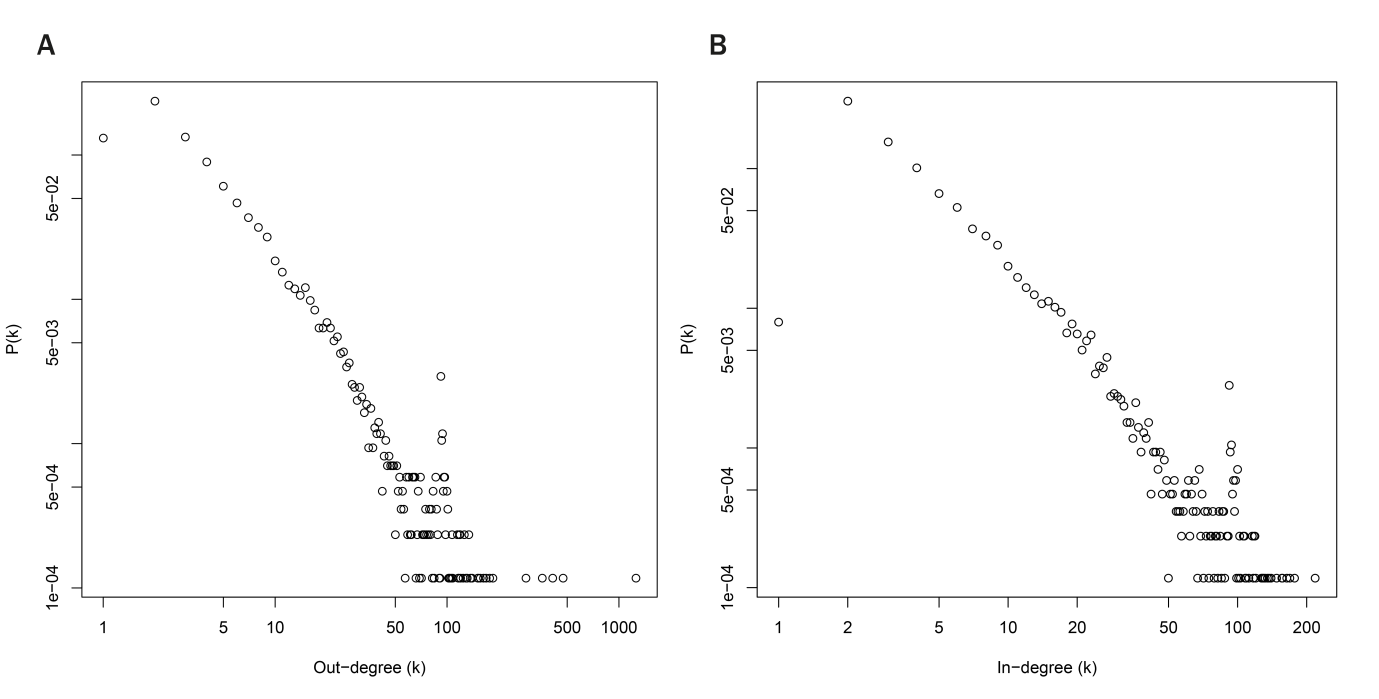


**Figure S6.** **Scale-freeness of a bridge network.** Degree distributions of a bridge network involved in bile acid metabolism by in-degrees (**A**) and out-degrees (**B**). The bile acid bridge network showed scale-free characteristics. Degree-distributions were determined separately, because the bridge network is a directed network.


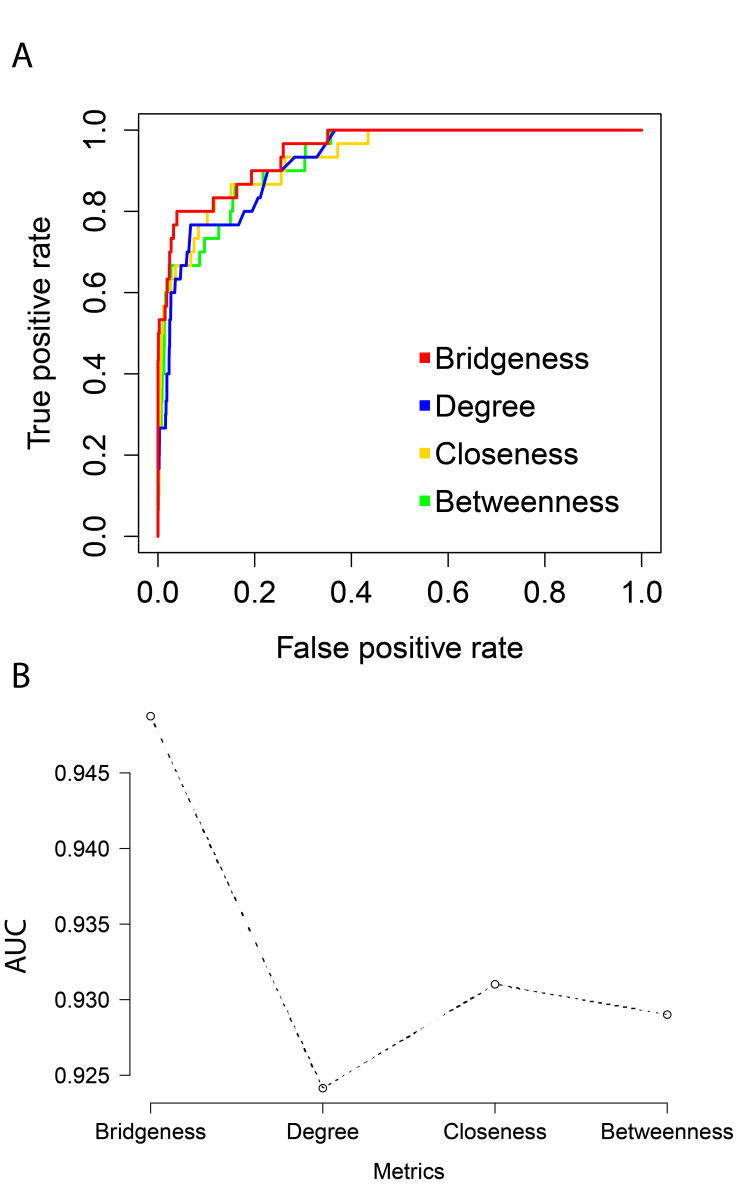


**Figure S7.** **Comparisons of centralities’ prediction accuracies.** Prediction accuracies were determined while predicting proteins on the shortest paths between a sensor and enzymes in a bile acid bridge network. (**A**) ROC curves according to type of centrality applied while predicting proteins. (**B**) AUC values of ROC curves in (**A**). Bridgeness could predict proteins on the shortest paths with the highest AUC value.

**Supporting Information Tables**

**Table S6. Top-50 bridge proteins with their network features such as shortest path info, degree, closeness, and betweenness**

| Bridgeness Rank | Entrez ID | Official gene name | On the shortest paths | Number of passing shortest paths | Bridgeness | Degree | Closeness | Betweenness | Degree Rank | Closeness Rank | Betweenness Rank |
| --- | --- | --- | --- | --- | --- | --- | --- | --- | --- | --- | --- |
| 1 | 10891 | PPARGC1A | Yes | 10 | 0.931924 | 0.007416916 | 0.228066751 | 0.00111984 | 479.5 | 733.5 | 1134 |
| 2 | 3172 | HNF4A | Yes | 8 | 0.858635 | 0.044644131 | 0.266132706 | 0.038561336 | 6 | 6 | 18 |
| 3 | 2932 | GSK3B | Yes | 2 | 0.842094 | 0.016260163 | 0.251831897 | 0.01305606 | 184.5 | 32 | 74 |
| 4 | 6258 | RXRG | Yes | 1 | 0.837569 | 0.003708458 | 0.217658564 | 0.000419908 | 1225 | 1800 | 2150 |
| 5 | 841 | CASP8 | Yes | 5 | 0.831109 | 0.016830695 | 0.243767602 | 0.009720026 | 176 | 86 | 108 |
| 6 | 1387 | CREBBP | Yes | 1 | 0.829978 | 0.044216232 | 0.259493671 | 0.037951439 | 8 | 14 | 19 |
| 7 | 5465 | PPARA | Yes | 1 | 0.826804 | 0.007987448 | 0.235719329 | 0.002124299 | 435 | 273 | 621 |
| 8 | 7157 | TP53 | Yes | 2 | 0.819395 | 0.073883897 | 0.276830135 | 0.141442327 | 2 | 2 | 1 |
| 9 | 2033 | EP300 | Yes | 1 | 0.814898 | 0.046355727 | 0.262495788 | 0.035833646 | 5 | 8 | 20 |
| 10 | 6256 | RXRA | Yes | 1 | 0.813953 | 0.016260163 | 0.2466404 | 0.008779531 | 184.5 | 63 | 123 |
| 11 | 2597 | GAPDH | Yes | 2 | 0.809105 | 0.014548567 | 0.239987677 | 0.009979943 | 204.5 | 143 | 106 |
| 12 | 8554 | PIAS1 | Yes | 4 | 0.807768 | 0.020253887 | 0.246623048 | 0.010229449 | 139.5 | 64 | 102 |
| 13 | 6670 | SP3 | Yes | 3 | 0.803686 | 0.007416916 | 0.247922487 | 0.001459615 | 479.5 | 53 | 897 |
| 14 | 5970 | RELA | No | 0 | 0.800425 | 0.036514049 | 0.261263276 | 0.031685374 | 16 | 10 | 25 |
| 15 | 3725 | JUN | No | 0 | 0.794695 | 0.03038083 | 0.261829182 | 0.023587039 | 31 | 9 | 38 |
| 16 | 7068 | THRB | No | 0 | 0.791072 | 0.009556411 | 0.236570387 | 0.002347138 | 339 | 245 | 553 |
| 17 | 1655 | DDX5 | No | 0 | 0.790658 | 0.00570532 | 0.245345745 | 0.001042353 | 721.5 | 75 | 1191 |
| 18 | 3660 | IRF2 | No | 0 | 0.790308 | 0.003708458 | 0.237475866 | 0.000531459 | 1225 | 210 | 1985 |
| 19 | 1487 | CTBP1 | No | 0 | 0.78389 | 0.007702182 | 0.226878519 | 0.003502604 | 454.5 | 828 | 345 |
| 20 | 7329 | UBE2I | Yes | 1 | 0.78364 | 0.022250749 | 0.2504018 | 0.025906336 | 126.5 | 41 | 32 |
| 21 | 2353 | FOS | No | 0 | 0.782764 | 0.016545429 | 0.25424282 | 0.009443283 | 182 | 28 | 115 |
| 22 | 6927 | HNF1A | Yes | 1 | 0.782106 | 0.01141064 | 0.242042395 | 0.006747448 | 265 | 101 | 166 |
| 23 | 4691 | NCL | No | 0 | 0.778803 | 0.010840108 | 0.233848104 | 0.003609732 | 288.5 | 351.5 | 334 |
| 24 | 1958 | EGR1 | No | 0 | 0.77681 | 0.00570532 | 0.240465084 | 0.001365858 | 721.5 | 133 | 959 |
| 25 | 602 | BCL3 | No | 0 | 0.776026 | 0.006418485 | 0.23745978 | 0.00129387 | 596 | 211.5 | 993 |
| 26 | 10514 | MYBBP1A | No | 0 | 0.77602 | 0.004849522 | 0.228007415 | 0.000547941 | 885.5 | 738 | 1963 |
| 27 | 2551 | GABPA | No | 0 | 0.774769 | 0.002567394 | 0.224230019 | 0.000398065 | 1703.5 | 1074 | 2189 |
| 28 | 96764 | TGS1 | No | 0 | 0.771331 | 0.002567394 | 0.217422316 | 0.000132699 | 1703.5 | 1824 | 3029 |
| 29 | 1936 | EEF1D | No | 0 | 0.770337 | 0.005420054 | 0.228430861 | 0.004582386 | 774.5 | 703 | 251 |
| 30 | 5757 | PTMA | No | 0 | 0.769629 | 0.007987448 | 0.233832505 | 0.003686149 | 435 | 354 | 324 |
| 31 | 387082 | SUMO4 | No | 0 | 0.768716 | 0.017401227 | 0.236100354 | 0.014942073 | 169.5 | 263 | 62 |
| 32 | 5468 | PPARG | No | 0 | 0.766806 | 0.007559549 | 0.228900715 | 0.001092801 | 464 | 671 | 1152 |
| 33 | 6517 | SLC2A4 | Yes | 1 | 0.765782 | 0.009413778 | 0.224222848 | 0.008373163 | 347.5 | 1077 | 128 |
| 34 | 405 | ARNT | No | 0 | 0.763764 | 0.005420054 | 0.225245775 | 0.002532296 | 774.5 | 986 | 506 |
| 35 | 5451 | POU2F1 | No | 0 | 0.7625 | 0.008700613 | 0.236682196 | 0.003047585 | 387.5 | 240 | 400 |
| 36 | 220988 | HNRNPA3 | No | 0 | 0.761382 | 0.00855798 | 0.222183489 | 0.000170042 | 396.5 | 1334 | 2860 |
| 37 | 6722 | SRF | No | 0 | 0.76017 | 0.012979603 | 0.241475511 | 0.00760652 | 230 | 109 | 138 |
| 38 | 6938 | TCF12 | No | 0 | 0.760074 | 0.003993724 | 0.234348364 | 0.00203976 | 1130 | 337 | 645 |
| 39 | 3939 | LDHA | No | 0 | 0.759313 | 0.010554842 | 0.230102727 | 0.001859789 | 300.5 | 579 | 703 |
| 40 | 468 | ATF4 | No | 0 | 0.758784 | 0.004706889 | 0.232106204 | 0.001231233 | 915 | 437.5 | 1027 |
| 41 | 2908 | NR3C1 | No | 0 | 0.758197 | 0.022250749 | 0.251660146 | 0.013997191 | 126.5 | 35 | 66 |
| 42 | 6794 | STK11 | No | 0 | 0.758188 | 0.004849522 | 0.225877122 | 0.000974237 | 885.5 | 923 | 1245 |
| 43 | 3192 | HNRNPU | No | 0 | 0.7578 | 0.013692769 | 0.239872725 | 0.007732832 | 215 | 150 | 137 |
| 44 | 10540 | DCTN2 | No | 0 | 0.756344 | 0.001996862 | 0.204964041 | 0.001200851 | 2150.5 | 3150 | 1050 |
| 45 | 7155 | TOP2B | No | 0 | 0.755395 | 0.005847953 | 0.233606557 | 0.000604511 | 694.5 | 359.5 | 1722 |
| 46 | 4666 | NACA | No | 0 | 0.753893 | 0.001996862 | 0.214876793 | 0.000336504 | 2150.5 | 2057.5 | 2318 |
| 47 | 1499 | CTNNB1 | No | 0 | 0.752596 | 0.038796177 | 0.258403361 | 0.050942248 | 12 | 17 | 7 |
| 48 | 4654 | MYOD1 | No | 0 | 0.750353 | 0.011695906 | 0.241417307 | 0.006168734 | 258 | 111 | 178 |
| 49 | 1052 | CEBPD | No | 0 | 0.749937 | 0.00285266 | 0.223102625 | 0.000172645 | 1552 | 1215 | 2832 |
| 50 | 3065 | HDAC1 | No | 0 | 0.74718 | 0.033661389 | 0.249839641 | 0.022983692 | 22 | 45 | 41 |

**Table S7. Proteins that are on the shortest paths between a bile acid sensor and bile acid enzymes.**

| Official gene name | Entrez ID | Number of passing shortest paths | Description |
| --- | --- | --- | --- |
| PPARGC1A | 10891 | 10 | peroxisome proliferator-activated receptor gamma, coactivator 1 alpha |
| HNF4A | 3172 | 8 | hepatocyte nuclear factor 4, alpha |
| CASP8 | 841 | 5 | caspase 8, apoptosis-related cysteine peptidase |
| PIAS1 | 8554 | 4 | protein inhibitor of activated STAT, 1 |
| SP3 | 6670 | 3 | Sp3 transcription factor |
| CAV1 | 857 | 2 | caveolin 1, caveolae protein, 22kDa |
| GAPDH | 2597 | 2 | glyceraldehyde-3-phosphate dehydrogenase |
| GSK3B | 2932 | 2 | glycogen synthase kinase 3 beta |
| TP53 | 7157 | 2 | tumor protein p53 |
| APOA1 | 335 | 1 | apolipoprotein A-I |
| CREBBP | 1387 | 1 | CREB binding protein |
| EP300 | 2033 | 1 | E1A binding protein p300 |
| FHIT | 2272 | 1 | fragile histidine triad |
| NR5A2 | 2494 | 1 | nuclear receptor subfamily 5, group A, member 2 |
| HSF4 | 3299 | 1 | heat shock transcription factor 4 |
| ING2 | 3622 | 1 | inhibitor of growth family, member 2 |
| PPARA | 5465 | 1 | peroxisome proliferator-activated receptor alpha |
| MAPK3 | 5595 | 1 | mitogen-activated protein kinase 3 |
| RB1 | 5925 | 1 | retinoblastoma 1 |
| RXRA | 6256 | 1 | retinoid X receptor, alpha |
| RXRG | 6258 | 1 | retinoid X receptor, gamma |
| ATXN1 | 6310 | 1 | ataxin 1 |
| SCP2 | 6342 | 1 | sterol carrier protein 2 |
| SLC2A4 | 6517 | 1 | solute carrier family 2 (facilitated glucose transporter), member 4 |
| STAT1 | 6772 | 1 | signal transducer and activator of transcription 1, 91kDa |
| TAF1 | 6872 | 1 | TAF1 RNA polymerase II, TATA box binding protein (TBP)-associated factor, 250kDa |
| HNF1A | 6927 | 1 | HNF1 homeobox A |
| UBE2I | 7329 | 1 | ubiquitin-conjugating enzyme E2I |
| RECQL5 | 9400 | 1 | RecQ protein-like 5 |
| ITGB3BP | 23421 | 1 | integrin beta 3 binding protein (beta3-endonexin) |
| SMURF2 | 64750 | 1 | SMAD specific E3 ubiquitin protein ligase 2 |
